# Polymorphisms in the Th17 cell-related *RORC* gene are associated with spontaneous clearance of HCV in Chinese women

**DOI:** 10.1186/s12879-018-3153-2

**Published:** 2018-06-04

**Authors:** Zhe Xie, Yuantao Li, Lu Long, Hua Liang, Weiping Cai, Tao Shen

**Affiliations:** 1 Department of Microbiology and Center of Infectious Diseases, School of Basic Medical Sciences, Peking University, Beijing, 100191 China; 2 State Key laboratory of Infectious Disease Prevention and Control, National Center for AIDS/STD Control and Prevention, China CDC, Collaborative Innovation Center for Diagnosis and Treatment of Infectious Diseases, Beijing, China; 3 Department of Infectious Diseases, Guangzhou Eighth People’s Hospital, Guangzhou Medical University, Guangzhou, 510060 Guangdong China

**Keywords:** HCV, IFNL3, Polymorphism, RORC, Spontaneous clearance

## Abstract

**Background:**

Female gender and favorable *IFNL3* genotypes are the primary independent predictors of spontaneous clearance of HCV infection. However, chronic hepatitis C infection occurs in numerous women carrying favorable *IFNL3* genotypes, indicating that other host and/or virological factors contribute to the prognosis of infection.

**Methods:**

A cohort of 137 anti-HCV-positive female Han Chinese cases, including 64 chronic HCV carriers and 73 HCV spontaneous resolvers, was recruited in the study. 111 SNPs in 23 genes encoding HCV co-receptors, transcription factors, Toll-like receptors, co-stimulating molecules, and cytokines were selected for SNP analysis.

**Results:**

After comparison of genotypes and allelotype frequencies of 111 SNPs in 23 genes in the primary cohort, the SNPs rs9826 (*P* = 0.024 for CC/TT/CT; *P* = 0.015 for C allele/T allele) and rs1521177 (*P* = 0.017 for GG/TT/GT; *P* = 0.006 for G allele/T allele) in the *RORC* gene were significantly associated with spontaneous HCV clearance. In the sub-cohort carrying favorable *IFNL3* genotypes (rs12979860CC, rs8099917 TT, rs12980275 AA), rs1521177 (genotype: *P =* 0.040; allelotype: *P =* 0.021) remained significantly associated with spontaneous HCV clearance. Importantly, the most common *RORC* haplotype rs9826-T/rs1521177-T was presented at significantly different frequencies in resolvers and carriers in both the primary cohort (*P* = 0.0027) and the *IFNL3* favorable sub-cohort (*P =* 0.0117).

**Conclusions:**

This study indicates that genetic polymorphisms in human Th17-related *RORC* gene are associated with different natural prognosis of HCV infection. The *RORC* haplotype, rs9826-T/rs1521177-T, was favorable for spontaneous clearance of HCV infection.

**Electronic supplementary material:**

The online version of this article (10.1186/s12879-018-3153-2) contains supplementary material, which is available to authorized users.

## Background

Chronic hepatitis C infection is a global healthcare burden and associated with the development of liver cirrhosis, hepatocellular carcinoma, and death if untreated [1]. In China, the prevalence of HCV in the general population is estimated as 1.6% [2, 3], which equals to approximately 20 million HCV carriers. A minority of acutely infected individuals (approximately 15–40%) will resolve their infection and recover spontaneously [4]. It is reported that among host factors, gender, age, *IFNL3* (previously *IL28B*) genotypes, *KIR/HLA* alleles, ethnicity, and HIV co-infection are associated with spontaneous recovery [5–15]. In particular, female sex and favorable *IFNL3* genotypes (rs12979860 CC, rs8099917 TT and rs12980275 AA) are primary independent predictors of spontaneous clearance [5–9, 12, 13, 16, 17]. In the general Han Chinese ethnic population, the three favorable *IFNL3* genotypes are very common (~ 90%) [12, 16–18]. However, many females carrying favorable *IFNL3* genotypes suffer from chronic hepatitis C in China, indicating that besides *IFNL3* genotypes and possibly some *KIR/HLA* alleles, other immune-related factors associated with HCV spontaneous clearance still remain unknown.

T helper 17 cells (Th17), a subset of pro-inflammatory T helper cells, play an important role in adaptive immunity, protecting hosts against pathogens primarily through secreting cytokines(such as IL-17A, IL-17F, IL-21, and IL-22) [19]. Receptor retinoic acid-related orphan receptor C (RORC), a DNA-binding transcription factor, is the master regulator of Th17 differentiation [20, 21]. Th17 cells may have dual roles (both harmful and beneficial) in HCV-related disease [22]. Specific HCV-Th17 cells are involved in immune response modulation, and their levels are associated with severity of fibrosis and intrahepatic inflammatory status [23, 24]. On the other hand, several reports indicate that elevated Th17 responses could be associated with spontaneous HCV clearance [25, 26].

In this study, two single nucleotide polymorphisms (SNPs) located in the *RORC* gene were identified to be associated with spontaneous clearance of HCV infection.

## Methods

### Participants

In the present study, 190 HBsAg-negative, anti-HCV-positive, Han Chinese females were identified by screening a total of 1252 residents (80% of the local population) in Wangying Village, Shangcai County, Henan province, in August 2009. More than 90% of patients were former plasma donors, and the remaining individuals composed of their parents, spouses, or children. Samples were tested for anti-HCV antibody using the Architech anti-HCV system (Abbott Diagnostics, USA), and those with signal/cut-off ratios between 1.0 and 5.0 were confirmed by RIBA assay (HCV BLOT 3.0, MP diagnostics, USA). Plasma HCV viral load was determined using the Abbott RealTime HCV Amplification Kit (Abbott Molecular Inc., USA) according to the manufacturer’s instructions. Spontaneous resolvers were defined as subjects who were positive for HCV antibody and negative for plasma HCV RNA with no history of HCV-specific treatment. HCV RNA-negative status was confirmed in a second sample collected in 2012 and/or 2013. Chronic HCV carriers were defined as those patients positive for both HCV antibody and plasma HCV viral load and also confirmed by the follow-up detection. No participants had received any type of HCV-specific antiviral therapy. All subjects were screened for HIV-1 infection status. Anti-HIV-1 antibody status was initially tested by ELISA assay (GBI biotech Co., Ltd., Beijing, China) and confirmed by HIV Blot 2.2 WB assay (Genelabs Diagnostics, Singapore). All HIV-positive patients had received first-line antiretroviral therapy for 6~ 8 years, regularly or intermittently. None of the participants received any types of anti-HCV treatment.

**Fig. 1:**
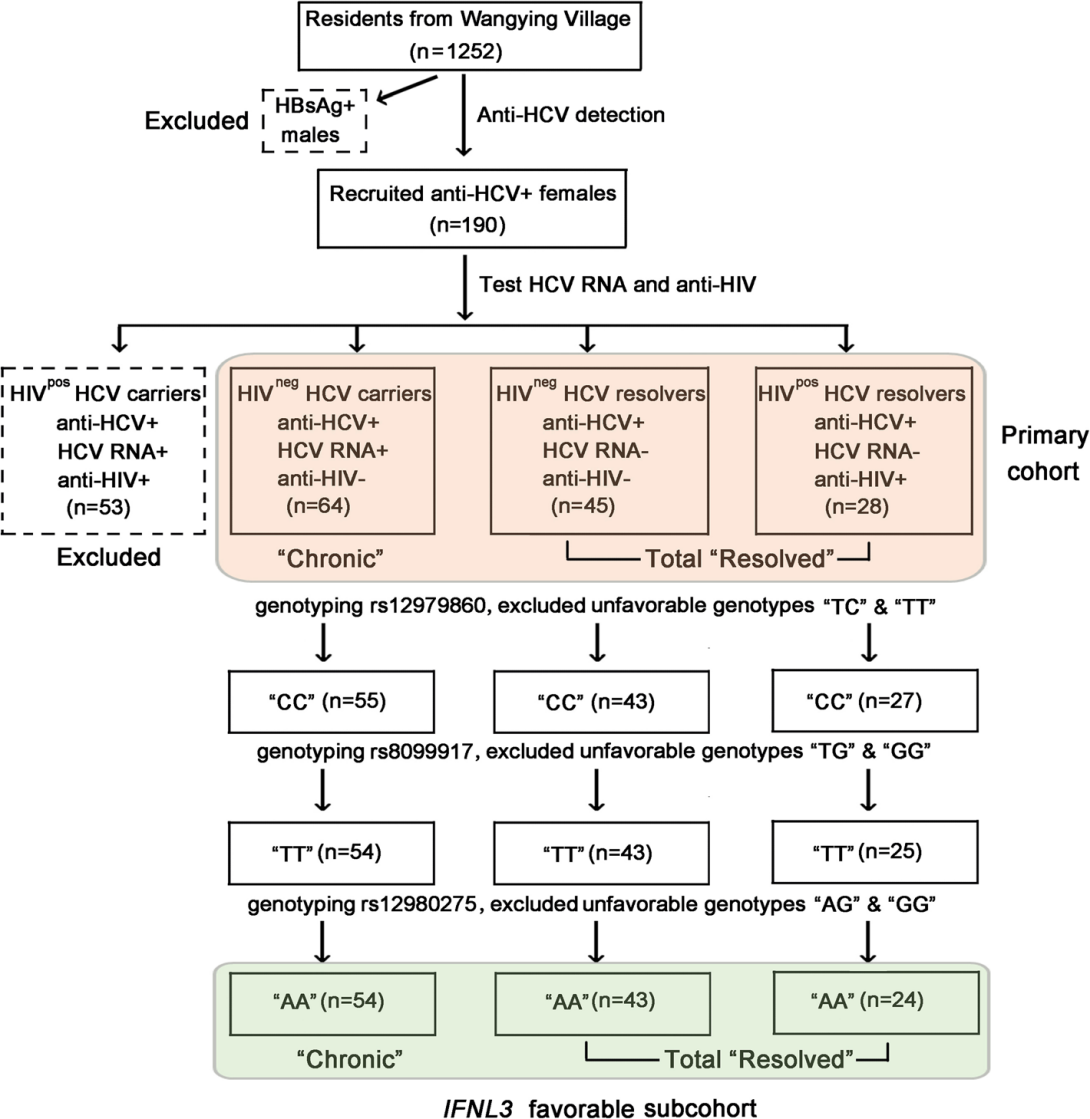
Flow diagram of study cohort selection. From a total of 1252 residents of Wangying Village, Shangcai County, 190 anti-HCV-positive female Chinese Han individuals were initially recruited to the study after screening. Of these 190 subjects, 53 HIV-positive women with chronic HCV infections were excluded. The remaining 137 females constituted the primary cohort, which consisted of “Chronic” (HIV- HCV carriers, *n* = 64) and “Resolved” (total *n* = 73: HIV+ HCV resolvers, *n* = 28, and HIV- HCV resolvers, *n* = 45) groups. An *IFNL3* favorable sub-cohort was also constructed by exclusion of women with unfavorable *IFNL3* genotypes (rs12979860 TC/TT, rs80999917 TG/TT, and rs12980275AG/GG; “Chronic”, *n* = 54, and “Resolved”, *n* = 67)

Of 190 female anti-HCV-positive individuals, HIV^pos^ women with chronic HCV infection (*n* = 53) were excluded from the study, as HIV infection could affect the ability of some individuals to spontaneously resolve their HCV infection. The final case number of primary cohort was 137, which consisted of a “Chronic” group (HIV^neg^ HCV carriers, *n* = 64) and a “Resolved” group (*n* = 73, including 45 HIV^neg^ HCV resolvers and 28 HIV^pos^ HCV resolvers). Overall, the cohort exhibited a similar high frequency of *IFNL3* favorable genotypes to Han Chinese nationality (Additional file 1: Table S1), indicating that the study was based on a representative cohort of the Chinese population [12, 16]. To eliminate the influence of *IFNL3* SNPs on viral clearance, a sub-cohort carrying favorable *IFNL3* genotypes (rs12979860CC, rs8099917TT, rs12980275 AA) was constructed and analyzed. The *IFNL3* favorable sub-cohort included 54 and 67 participants in the “Chronic” and “Resolved” (HIV^neg^, *n* = 43; HIV^pos^, *n* = 24) groups, respectively. A flow diagram for cohort construction was provided as Fig 1. The clinical and biochemical characteristics of the primary cohort were presented in Table 1.

**Table 1:** Characteristics of individuals in the primary cohort

Characteristic	Chronic HCV (*n* = 64)	Resolved HCV (n = 73)	
		HIV^neg^ (*n* = 45)	HIV^pos^ (*n* = 28)
Age (years)^a^	50 (47–59)	59 (49–67)	54 (47–64)
BMI^a^	23.5 (21.1–26.1)	23.2 (21.3–26.3)	22.1 (20.5–24.5)
Anti-HCV	Positive	Positive	Positive
Anti-HIV	Negative	Negative	Positive
HCV RNA (log_10_ IU/mL)^a^	6.17 (5.82–6.54)	Negative	Negative
HCV genotype			
1b (n,%)^b^	39 (60.9%)	–	–
2a (n,%)^b^	25 (39.1%)	–	–
Others	None	–	–
ALT (n,%)			
> 40 (IU/L)^b^	28 (43.7%)	4 (8.9%)	7 (25.0%)
≤40 (IU/L)^b^	36 (56.3%)	41 (91.1%)	21 (75.0%)
AST (n,%)			
> 40 (IU/L)^b^	30 (46.9%)	1 (2.2%)	7 (25.0%)
≤40 (IU/L)^b^	34 (53.1%)	44 (97.8%)	21 (75.0%)
CD4+ T cells/μL^a^	882 (599–1088)	888 (658–1119)	445 (346–587)

Data presented as ^a^medians (inter-quartile range) or ^b^number of cases (%)

*BMI* body mass index, *ALT* alanine aminotransferase, *AST* aspartate aminotransferase

### SNP selection and genotyping

A total of 111 SNPs in 23 genes encoding HCV co-receptors (*CD81*, *SCARB1*, *CLDN1*, *OCLN*, *NPC1L1*, *APOE*, *LDLR*), transcription factors(*IRF3*, *RORC*, *TBX21*, *FOXP3*, *BCL6*), Toll-like receptors (*TLR3*, *TLR7*, *TLR9*), co-stimulating molecules (*ICOS*, *CXCR5*, *CD40LG*), and cytokines(*IL4*, *IFNG*, *IL21*, *IFNL3*, *CXCL13*) were selected for SNP analysis (Additional file 1: Table S2). The selected SNPs met at least one of the following criteria:1) a reported minor allele frequency > 10% in the Han Chinese population according to SNP browser software 4.0 (Applied Biosystems), with reference to the NCBI SNP database (http://www.ncbi.nlm.nih.gov/projects/SNP/); 2) published evidence that the SNP was associated with disease.

Genomic DNA extracted from whole blood samples using a DNeasy Blood & Tissue kit (Qiagen, USA) was dissolved in sterile double distilled water and stored at − 20 °C until use. DNA purity was checked and DNA samples with 260/280 ratios< 1.7 were re-purified. SNP genotyping was performed using the iPLEX Sequenom MassARRAY system (Sequenom Inc., USA).

### Statistical analyses

Statistical and graphical analyses were performed using GraphPad Prism 5.0, Microsoft Excel 2007, or SPSS 20.0. The allele frequency and genotype distributions of each SNP were descriptively summarized as numbers of cases and frequencies. Chi-square (χ^2^) and Fisher’s exact tests were used to examine differences in frequencies of individual SNPs between HCV carriers and spontaneous resolvers. SNP-specific deviation from Hardy–Weinberg Equilibrium (HWE) in the whole study population was tested using a χ^2^ test in SHEsis software, and SNPs with HWE violation (*P* < 0.01) were excluded. Pairwise D′ and r^2^ measures of linkage disequilibrium for *RORC* SNPs were calculated using SHEsis (http://analysis.bio-x.cn/myAnalysis.php). Specific parameters were set as previously described [27]. *P*-values, odds ratios (ORs), and 95% confidence intervals (95% CIs) were used for association analysis. All *P*-values were two-tailed, and were considered significant at < 0.05.

## Results

### Associations between target gene SNPs and spontaneous HCV clearance in the primary cohort

Genotype distributions and allele frequencies were calculated for 111 candidate SNPs in both the “Chronic” and “Resolved” groups of the primary cohort (Additional file 1: Table S2). The frequencies of favorable *IFNL3* genotypes were higher in “Resolved” group than “Chronic” group (resolved vs. chronic: rs12979860, CC 96% vs. 86%; rs8099917, TT 95% vs. 91%; and rs12980275, AA 96% vs. 86%), although only the difference in the frequency of the rs12979860 C allele was statistically significant (*P* = 0.043) (Table 2).

**Table 2:** Genotype and allele frequency distributions of *IFNL3*and *RORC*SNPs in the primary cohort

SNP	Genotype	Chronic HCV^a^(*n* = 64)	Resolved HCV^a^(*n* = 73)	*P*-value	OR (95% CI)
rs12979860	CC	55 (0.85)	70(0.96)	0.105	
	TT	1 (0.02)	0 (0.00)		
	CT	8 (0.13)	3 (0.04)		
	C allele	118 (0.92)	143 (0.98)	**0.043**	0.25 (0.07–0.87)
	T allele	10 (0.08)	3 (0.02)		4.04 (1.15–13.89)
rs8099917	TT	58(0.91)	69 (0.95)	0.514	
	GG	0 (0.00)	0 (0.00)		
	GT	6 (0.09)	4 (0.05)		
	T allele	122 (0.95)	142 (0.97)	0.523	0.57 (0.16–2.08)
	G allele	6 (0.05)	4 (0.03)		1.75 (0.48–6.33)
rs12980275	AA	55 (0.85)	69 (0.96)	0.189	
	GG	1 (0.02)	0 (0.00)		
	AG	8 (0.13)	4 (0.04)		
	A allele	118 (0.92)	142 (0.97)	0.096	0.33 (0.10–1.09)
	G allele	10 (0.08)	4 (0.03)		3.01 (0.92–9.84)
*RORC* gene					
rs9826	CC	7 (0.11)	1 (0.01)	**0.024**	
(3′ UTR)	TT	29 (0.45)	45 (0.62)		
	CT	28 (0.44)	27 (0.37)		
	C allele	42 (0.33)	29 (0.20)	**0.015**	1.97 (1.14–3.41)
	T allele	86 (0.67)	117 (0.80)		0.51 (0.29–0.88)
rs1521177	GG	6 (0.10)	1 (0.01)	**0.017**	
(intron)	TT	29 (0.45)	48 (0.66)		
	GT	29 (0.45)	24 (0.33)		
	G allele	41 (0.32)	26 (0.18)	**0.006**	2.18 (1.24–3.82)
	T allele	87 (0.68)	120 (0.82)		0.46 (0.26–0.81)

^a^Number of cases (frequency). *SNP* genotypes were determined using the iPLEX MassARRAY system (Sequenom Inc., USA), and allele frequencies and genotype distributions were calculated. Chi-square (χ^2^) and Fisher’s exact tests were used to evaluate differences in SNP frequencies between HCV carriers and spontaneous resolvers. *P*-values, odds ratios, (*ORs*), and 95% confidence intervals, (*95% CIs*) were determined for association analysis. *P*-values (two-tailed) < 0.05 were considered significant (bold)

Of the 22 remaining genes tested (excluding *IFNL3*), only SNPs rs9826 and rs1521177, located in non-coding regions of *RORC*, exhibited frequency differences between the “Resolved” and “Chronic” groups (rs9826: *P* = 0.024 for CC/TT/CT, *P* = 0.015 for C allele/T allele, OR = 1.97, 95% CI: 1.14-3.41; rs1521177: *P* = 0.017 for GG/TT/GT, *P* = 0.006 for G allele/T allele, OR = 2.18, 95% CI: 1.24-3.82)(Table 2). The rs9826 TT and rs1521177 TT genotypes were associated with improved viral clearance. Overall, the primary cohort exhibited a similar distribution of *RORC* genotypes to Han Chinese nationality (Additional file 1: Table S3). The Hardy–Weinberg Equilibrium (HWE) test of three *IFNL3* SNPs and two *RORC* SNPs in the primary cohort were tested, and no SNPs with HWE violation were presented in the study (Additional file 1: Table S4).

In the present study, a minority of “Resolved” individuals were co-infected with HIV (HIV^pos^ resolved, *n* = 28) and very similar *IFNL3* (rs12979860, rs8099917, and rs12980275) and *RORC* (rs9826 and rs1521177) SNP genotype distributions were identified among HIV^neg^, HIV^pos^, and total resolved individuals (Additional file 1: Figure S1). No association was observed between RORC SNPs (rs9826 and rs1521177) and HCV RNA levels in HIV-uninfected “Chronic” individuals (Additional file 1: Figure S2). In addition, we did not find any differences in genotypes and allele frequency distributions of *RORC* SNPs (rs9826 and rs1521177) between HIV^pos^ HCV carriers and HIV^pos^ HCV resolvers (Additional file 1: Table S5), and no associations between *RORC* SNPs and type of HCV genotypes (HCV 2a vs. HCV 1b) was found in HIV^neg^ HCV carriers (Additional file 1: Table S6).

### Re-evaluation of RORC SNP associations with spontaneous HCV clearance in the IFNL3 favorable sub-cohort

In the *IFNL3* favorable sub-cohort, cases with unfavorable *IFNL3* genotypes in the “Chronic” (*n* = 10) and “Resolved” (*n* = 6) groups were excluded. Analysis of the sub-cohort indicated that significant differences between the two groups remained for rs1521177 (*P* = 0.040 for GG/TT/GT, *P* = 0.021 for G allele/T allele, OR = 2.00, 95% CI: 1.11-3.55), although no significant differences were identified for rs9826 (*P* = 0.092 for CC/TT/CT, *P* = 0.062 for C allele/T allele, OR = 1.74, 95% CI: 0.97-3.19) (Table 3). These data suggested that SNP alleles in *RORC* had an independent effect on HCV viral clearance, which couldnot be explained by the distribution of *IFNL3* polymorphisms.

**Table 3:** Genotype and allele distributions of SNPs in the *RORC* gene in the *IFNL3* favorable sub-cohort^a^

*RORC* SNPs	Chronic HCV^b^ (*n* = 54)	Resolved HCV^b^ (*n* = 67)	*P*-value	OR (95% CI)
rs9826				
CC	5 (0.09)	1 (0.01)	0.092	
TT	26 (0.48)	41 (0.61)		
CT	23 (0.43)	25 (0.37)		
C allele	33 (0.31)	27 (0.20)	0.062	1.74 (0.97–3.19)
T allele	75 (0.69)	107 (0.80)		0.57 (0.31–1.03)
rs1521177				
GG	6 (0.11)	1 (0.01)	**0.040**	
TT	26 (0.48)	43 (0.64)		
GT	22 (0.41)	23 (0.34)		
G allele	34 (0.31)	25 (0.19)	**0.021**	2.00 (1.11–3.55)
T allele	74 (0.69)	109 (0.81)		0.50 (0.28–0.90)

^a^Participants in the, *IFNL3* favorable sub-cohort were screened for, *IFNL3* genotypes (rs12979860CC/rs8099917TT/rs12980275AA)

^b^Number of cases (frequency)

*SNP* genotyping was conducted using the iPLEX MassARRAY system (Sequenom Inc., USA), and allele and genotype frequency distributions were calculated. Chi-square (χ^2^) and Fisher’s exact tests were used to evaluate differences in, *SNP* frequencies between, *HCV* carriers and spontaneous resolvers. *P*-values, odds ratios, (*ORs*) and 95% confidence intervals, (*95% CIs*) were determined for association analysis. *P*-values (two-tailed) < 0.05were considered significant (bold)

### Analysis of the RORCrs9826/rs1521177 haplotype

Linkage disequilibrium tests for *RORC* and *IFNL3* SNPs in the primary cohort were shown in Additional file 1: Figure S3. The *RORC* SNPs, rs9826 and rs1521177, were determined to be in linkage disequilibrium (Primary cohort: D’ = 0.831, r^2^ = 0.639; *IFNL3* favorable sub-cohort: D’ = 0.812, r^2^ = 0.645) (Additional file 1: Table S7). Two main haplotypes were present in the primary cohort (rs9826-T/rs1521177-T: 62% vs. 79%, chronic vs. resolved; rs9826-C/rs1521177-G: 27% vs. 16%, chronic vs. resolved) and the *IFNL3* favorable sub-cohort (rs9826-T/rs1521177-T: 64% vs. 78%, chronic vs. resolved; rs9826-C/rs1521177-G: 26% vs. 17%, chronic vs. resolved). Overall, the presence of the rs9826-T/rs1521177-T haplotype was associated with a higher likelihood of spontaneous resolution in both the primary cohort (*P* = 0.0027, OR = 0.445, 95% CI: 0.26–0.76) and the *IFNL3* favorable sub-cohort (*P* = 0.0117, OR = 0.485, 95% CI:0.28–0.86). In addition, the frequency of the rs9826-C/rs1521177-G haplotype was significantly different between the chronic and resolved groups in the primary cohort (*P* = 0.0315, OR = 1.893, 95% CI:1.05–3.40), suggesting that individuals carrying this haplotype was difficult in spontaneous resolution of HCV infection (Table 4).

**Table 4:** Common *RORC* SNP haplotypes (rs9826/rs1521177) and their association with spontaneous viral clearance in the primary cohort and *IFNL3* favorable sub-cohort

	Chronic HCV^a^	Resolved HCV^a^	*P*-value	OR (95% CI)
Primary cohort				
T/T	0.62	0.79	**0.0027**	0.445(0.26–0.76)
C/G	0.27	0.16	**0.0315**	1.893(1.05–3.40)
*IFNL3* favorable sub-cohort				
T/T	0.64	0.78	**0.0117**	0.485(0.28–0.86)
C/G	0.26	0.17	0.1042	1.672(0.90–3.12)

^a^Haplotype frequency. Chi-square (χ^2^) tests were used to evaluate differences in haplotype frequencies between, *HCV* carriers and spontaneous resolvers, using SHEsis software. Haplotypes with frequencies < 0.05 (C/T, C/G, C/T, T/G) are not shown. *P*-values, odds ratios, (*ORs*) and 95% confidence intervals (*95% CIs*) were determined for association analysis. *P*-values (two-tailed) < 0.05 were considered significant (bold)

## Discussion

Genetic polymorphisms of innate immunity-related genes were reported to be associated with HCV clearance [28, 29]. Specially, SNPs of the *IFNL3* (IL-28B) have shown strong association with spontaneous clearance of HCV and with the response to anti-HCV therapy [30–33]. Another important correlation of HCV spontaneously viral clearance was a robust and sustained HCV-specific T cell response. Cumulative studies indicated that HLA-associated viral polymorphisms were closely correlated with immune recognition of T cells to virus (both HIV and HCV) [34–37].

Exploration of adaptive immune factors involved in spontaneous clearance of viral infection will help to understand HCV pathogenesis. Besides *IFNL* genotypes, HCV-specific T cells are typically detectable 5–9 weeks after acute infection [38, 39]. Recently, Grebely et al. reported that the median time for acute HCV clearance among 173 spontaneous resolvers undergoing follow-up observation was 16.5 weeks [9], indicating that a strong, broad, and persistent HCV-specific adaptive immune response is required during acute infection for viral clearance [40].

The role of Th17 responses in HCV pathogenesis is intriguing but not well characterized. Numerous studies have focused on its destructive potentials, since they could aggravate the severity of fibrosis and intrahepatic inflammatory status [22, 41, 42]. However, some studies have also reported a role for Th17 responses in spontaneous HCV clearance. Kared et al. reported that the differentiation of IL-17A-producing CD4+ T cells was correlated with prognosis of infection [26]. In addition, the concentration of plasma IL-17A was significantly higher in the acute phase of HCV infection in patients with self-limiting infection than in those with chronically evolving hepatitis [26]. Moreover, Seetharam et al. described a transient IL-17 response followed by a subsequent reactivation of Th1 responses, resulting to spontaneous recovery in a liver transplant recipient with recurrent hepatitis C [25]. It is possible that Th17 responses in HCV-associated disorders may act as a double-edged sword, playing different roles in different disease courses.

In this study, a number of the HCV resolvers had a background of HIV infection, which could be primarily ascribed to historical contaminated commercial blood donations in the late 1990s. As HCV is more efficiently transmitted through blood contamination or infected needles than HIV, self-limiting acute HCV infection in these individuals may usually precede, or be coincident with, HIV infection [6, 43, 44]. Regardless of whether spontaneous recovery from HCV occurred before or after HIV infection, it remains conceivable that genetic immune characteristics of these individuals could be associated with spontaneous HCV eradication. By contrast, we excluded chronically HIV/HCV-co-infected patients from the study, due to the possibility that some HIV-co-infected individuals who could clear HCV spontaneously in the absence of HIV became chronic HCV carriers. In addition, all the participants in this study came from the same village and shared similar characteristics of viral contamination mode, daily diet, surrounding environment, ethnicity, and income level, which strengthed the comparison of genetic immune factors between HCV carriers and resolvers. Among the two *RORC* SNPs (rs9826 and rs1521177) identified in this study, the rs9826 polymorphism was associated with severity of rheumatoid arthritis in the Polish population [45]. Further functional studies to explore the associations between *RORC* SNPs genotypes and Th17 responses, as well as Th17 responses and viral clearance, should be performed in the future.

Our cohort design precluded the influence factors of ethnicity, gender and IFNL-3. However, the sample size of our cohort was still limited, and future larger-scale queues were needed for better verification of our conclusion. In addition, the *RORC* SNPs rs9826-T and rs1521177-T were validated in only Chinese Han population in this study. The adaptation of these SNPs in other ethnic and national populations needs to be further analyzed.

## Conclusions

The present study determined that polymorphisms in human Th17-related *RORC* gene were associated with differing natural prognosis for Chinese Han females with HCV infection. The most common *RORC* haplotype in this cohort, rs9826-T/rs1521177-T, was favorable for spontaneous clearance of HCV infection.

## Additional file

Additional file 1: **Figure S1.** Comparison of distributions of *IFNL3* and *RORC* SNP genotypes among HIVneg, HIVpos, and total resolved individuals. (**a**) Distribution of genotypes (%) of three *IFNL3* SNPs (rs12979860, rs8099917, and rs12980275). (**b**) Distribution of genotypes (%) of two *RORC* SNPs (rs9826, and rs1521177). Chi-square (χ2) and Fisher’s exact tests were used to evaluate the differences in SNP distributions between two groups. *P*-values (two-tailed) < 0.05 were considered significant (n.s., not significant). **Figure S2.** HCV viral load levels of *RORC* SNP genotypes (rs9826, rs1521177) among HIV^neg^ HCV carriers of the primary cohort. **Figure S3.** Linkage disequilibrium tests for RORC SNPs (rs9826 and rs1521177) and *IFNL3* SNPs (rs12979860, rs8099917 and rs12980275) in the primary cohort (**a**) D’ value. (**b**) r^2^ value. **Table S1.** Allele frequencies of *IFNL3* gene SNPs in different populations. **Table S2.** Allele, genotype, and carrier frequencies and percentages of tested SNPs in the primary cohort. **Table S3.** *RORC* gene SNP allele frequencies in different populations. **Table S4.** The Hardy–Weinberg Equilibrium (HWE) test of all SNPs in the whole study population. **Table S5.** Genotype and allele frequency distributions of *RORC* SNPs in HIV^pos^ group. **Table S6.** Genotype and allele frequency distributions of *RORC* SNPs in HIV^neg^ HCV carriers of the primary cohort. **Table S7.** Linkage disequilibrium tests for *RORC* rs9826/rs1521177 in the primary cohort and the *IFNL3* favorable sub-cohort. (DOC 1671 kb)

## Abbreviations

95% CIs: 95% confidence intervals
HAART: High active antiretroviral therapy
HCC: Hepatocellular carcinoma
HCV: Hepatitis C virus
HIV: Human immunodeficiency virus
*IFNL3*: Interferon lambda 3
LD: Linkage disequilibrium
OR: Odds ratios
RORC: Receptor retinoic acid-related orphan receptor C
SNP: Single nucleotide polymorphisms
Th17: T helper 17 cells

## References

[1] Messina JP, Humphreys I, Flaxman A, Brown A, Cooke GS, Pybus OG, Barnes E (2015). Global distribution and prevalence of hepatitis C virus genotypes. Hepatology.

[2] Kao JH, Ahn SH, Chien RN, Cho M, Chuang WL, Jeong SH, Liu CH, Paik SW (2017). Urgency to treat patients with chronic hepatitis C in Asia. J Gastroenterol Hepatol.

[3] Bennett H, Waser N, Johnston K, Kao JH, Lim YS, Duan ZP, Lee YJ, Wei L, Chen CJ, Sievert W (2015). A review of the burden of hepatitis C virus infection in China, Japan South Korea and Taiwan. Hepatol Int.

[4] Mohd Hanafiah K, Groeger J, Flaxman AD, Wiersma ST (2013). Global epidemiology of hepatitis C virus infection: new estimates of age-specific antibody to HCV seroprevalence. Hepatology.

[5] Huang J, Huang K, Xu R, Wang M, Liao Q, Xiong H, Li C, Tang X, Shan Z, Zhang M (2016). The associations of HLA-A*02:01 and DRB1*11:01 with hepatitis C virus spontaneous clearance are independent of IL28B in the Chinese population. Sci Rep.

[6] van den Berg CH, Grady BP, Schinkel J, van de Laar T, Molenkamp R, van Houdt R, Coutinho RA, van Baarle D, Prins M (2011). Female sex and IL28B, a synergism for spontaneous viral clearance in hepatitis C virus (HCV) seroconverters from a community-based cohort. PLoS One.

[7] Alric L, Bonnet D, Fort M (2014). Association between female sex, IL28B genotype, but also DQB1*0301 allele and the outcome of acute hepatitis C virus infection. Hepatology.

[8] Ikezaki H, Furusyo N, Hiramine S, Ura K, Mitsumoto-Kaseida F, Takayama K, Shimizu M, Toyoda K, Ogawa E, Kainuma M (2016). Association of IL28B rs8099917 genotype and female sex with spontaneous clearance of hepatitis C virus infection: a Japanese cross-sectional study. Arch Virol.

[9] Grebely J, Page K, Sacks-Davis R, van der Loeff MS, Rice TM, Bruneau J, Morris MD, Hajarizadeh B, Amin J, Cox AL (2014). The effects of female sex, viral genotype, and IL28B genotype on spontaneous clearance of acute hepatitis C virus infection. Hepatology.

[10] Moqueet N, Infante-Rivard C, Platt RW, Young J, Cooper C, Hull M, Walmsley S, Klein MB (2015). Favourable IFNL3 genotypes are associated with spontaneous clearance and are differentially distributed in aboriginals in Canadian HIV-hepatitis C co-infected individuals. Int J Mol Sci.

[11] Peng J, Chen X, He J, Zheng J, Qin B, Jiang Y (2015). Relationship between interleukin 28B, equilibrative nucleoside transporters 1 gene polymorphisms and spontaneous clearance of HCV in HIV/HCV co-infectors. Zhonghua liu xing bing xue za zhi = Zhonghua liuxingbingxue zazhi.

[12] Shi X, Pan Y, Wang M, Wang D, Li W, Jiang T, Zhang P, Chi X, Jiang Y, Gao Y (2012). IL28B genetic variation is associated with spontaneous clearance of hepatitis C virus, treatment response, serum IL-28B levels in Chinese population. PLoS One.

[13] Rao HY, Sun DG, Jiang D, Yang RF, Guo F, Wang JH, Liu F, Zhang HY, Zhang HH, Du SC (2012). IL28B genetic variants and gender are associated with spontaneous clearance of hepatitis C virus infection. J Viral Hepat.

[14] Sagnelli E, Santantonio T, Coppola N, Fasano M, Pisaturo M, Sagnelli C (2014). Acute hepatitis C: clinical and laboratory diagnosis, course of the disease, treatment. Infection.

[15] Khakoo SI, Thio CL, Martin MP, Brooks CR, Gao X, Astemborski J, Cheng J, Goedert JJ, Vlahov D, Hilgartner M (2004). HLA and NK cell inhibitory receptor genes in resolving hepatitis C virus infection. Science.

[16] Dong ZX, Zhou HJ, Xiang XG, Guo SM, Zhuang Y, Zhao GD, Xie Q (2015). IL28B genetic variations are associated with treatment response of patients with chronic hepatitis C in a Chinese Han population. J Dig Dis.

[17] Jin G, Kang H, Chen X, Dai D (2014). Evaluation of the relationship between IL28B, IL10RB and IL28RA single-nucleotide polymorphisms and susceptibility to hepatitis C virus in Chinese Han population. Infec Genet Evol.

[18] Liao XW, Ling Y, Li XH, Han Y, Zhang SY, Gu LL, Yu DM, Yao BL, Zhang DH, Jin GD (2011). Association of genetic variation in IL28B with hepatitis C treatment-induced viral clearance in the Chinese Han population. Antivir Ther.

[19] Zambrano-Zaragoza JF, Romo-Martinez EJ, Duran-Avelar Mde J, Garcia-Magallanes N, Vibanco-Perez N (2014). Th17 cells in autoimmune and infectious diseases. Int J Inflamm.

[20] Benoit G, Cooney A, Giguere V, Ingraham H, Lazar M, Muscat G, Perlmann T, Renaud JP, Schwabe J, Sladek F (2006). International Union of Pharmacology LXVI. Orphan nuclear receptors. Pharmacol Rev.

[21] Sun Z, Unutmaz D, Zou YR, Sunshine MJ, Pierani A, Brenner-Morton S, Mebius RE, Littman DR (2000). Requirement for RORgamma in thymocyte survival and lymphoid organ development. Science.

[22] Balanescu P, Ladaru A, Voiosu T, Nicolau A, Ene M, Balanescu E (2012). Th17 and IL-17 immunity in chronic hepatitis C infection. Rom J Intern Med.

[23] Chang Q, Wang YK, Zhao Q, Wang CZ, Hu YZ, Wu BY (2012). Th17 cells are increased with severity of liver inflammation in patients with chronic hepatitis C. J Gastroenterol Hepatol.

[24] Sun HQ, Zhang JY, Zhang H, Zou ZS, Wang FS, Jia JH (2012). Increased Th17 cells contribute to disease progression in patients with HBV-associated liver cirrhosis. J Viral Hepat.

[25] Seetharam AB, Borg BB, Subramanian V, Chapman WC, Crippin JS, Mohanakumar T (2011). Temporal association between increased virus-specific Th17 response and spontaneous recovery from recurrent hepatitis C in a liver transplant recipient. Transplantation.

[26] Kared H, Fabre T, Bedard N, Bruneau J, Shoukry NH (2013). Galectin-9 and IL-21 mediate cross-regulation between Th17 and Treg cells during acute hepatitis C. PLoS Pathog.

[27] Chen J, Liang Z, Lu F, Fang X, Liu S, Zeng Y, Zhu F, Chen X, Shen T, Li J (2011). Toll-like receptors and cytokines/cytokine receptors polymorphisms associate with non-response to hepatitis B vaccine. Vaccine.

[28] Romero-Gomez M, Eslam M, Ruiz A, Maraver M (2011). Genes and hepatitis C: susceptibility, fibrosis progression and response to treatment. Liver Int.

[29] Selvarajah S, Tobler LH, Simmons G, Busch MP (2010). Host genetic basis for hepatitis C virus clearance: a role for blood collection centers. Curr Opin Hematol.

[30] Ge D, Fellay J, Thompson AJ, Simon JS, Shianna KV, Urban TJ, Heinzen EL, Qiu P, Bertelsen AH, Muir AJ (2009). Genetic variation in IL28B predicts hepatitis C treatment-induced viral clearance. Nature.

[31] Suppiah V, Moldovan M, Ahlenstiel G, Berg T, Weltman M, Abate ML, Bassendine M, Spengler U, Dore GJ, Powell E (2009). IL28B is associated with response to chronic hepatitis C interferon-alpha and ribavirin therapy. Nat Genet.

[32] Tanaka Y, Nishida N, Sugiyama M, Kurosaki M, Matsuura K, Sakamoto N, Nakagawa M, Korenaga M, Hino K, Hige S (2009). Genome-wide association of IL28B with response to pegylated interferon-alpha and ribavirin therapy for chronic hepatitis C. Nat Genet.

[33] Thomas DL, Thio CL, Martin MP, Qi Y, Ge D, O'Huigin C, Kidd J, Kidd K, Khakoo SI, Alexander G (2009). Genetic variation in IL28B and spontaneous clearance of hepatitis C virus. Nature.

[34] Gaudieri S, Rauch A, Park LP, Freitas E, Herrmann S, Jeffrey G, Cheng W, Pfafferott K, Naidoo K, Chapman R (2006). Evidence of viral adaptation to HLA class I-restricted immune pressure in chronic hepatitis C virus infection. J Virol.

[35] Ansari MA, Pedergnana V, LCI C, Magri A, Von Delft A, Bonsall D, Chaturvedi N, Bartha I, Smith D, Nicholson G (2017). Genome-to-genome analysis highlights the effect of the human innate and adaptive immune systems on the hepatitis C virus. Nat Genet.

[36] Moore CB, John M, James IR, Christiansen FT, Witt CS, Mallal SA (2002). Evidence of HIV-1 adaptation to HLA-restricted immune responses at a population level. Science.

[37] von Delft A, Humphreys IS, Brown A, Pfafferott K, Lucas M, Klenerman P, Lauer GM, Cox AL, Gaudieri S, Barnes E (2016). The broad assessment of HCV genotypes 1 and 3 antigenic targets reveals limited cross-reactivity with implications for vaccine design. Gut.

[38] Thimme R, Bukh J, Spangenberg HC, Wieland S, Pemberton J, Steiger C, Govindarajan S, Purcell RH, Chisari FV (2002). Viral and immunological determinants of hepatitis C virus clearance, persistence.and disease. Proc Nati Acad Sci USA.

[39] Thimme R, Oldach D, Chang KM, Steiger C, Ray SC, Chisari FV (2001). Determinants of viral clearance and persistence during acute hepatitis C virus infection. J Exp Med.

[40] Klenerman P, Thimme R (2012). T cell responses in hepatitis C: the good the bad and the unconventional. Gut.

[41] Basha HI, Subramanian V, Seetharam A, Nath DS, Ramachandran S, Anderson CD, Shenoy S, Chapman WC, Crippin JS, Mohanakumar T (2011). Characterization of HCV-specific CD4+Th17 immunity in recurrent hepatitis C-induced liver allograft fibrosis. Am J Transplant Off J Am Soc Transplant Am Soc Transplant Surg.

[42] Wang JM, Shi L, Ma CJ, Ji XJ, Ying RS, Wu XY, Wang KS, Li G, Moorman JP, Yao ZQ (2013). Differential regulation of interleukin-12 (IL-12)/IL-23 by Tim-3 drives T(H)17 cell development during hepatitis C virus infection. J Virol.

[43] Shen T, Chen X, Zhang W, Xi Y, Cao G, Zhi Y, Wang S, Xu C, Wei L, Lu F (2011). A higher correlation of HCV core antigen with CD4+ T cell counts compared with HCV RNA in HCV/HIV-1 coinfected patients. PLoS One.

[44] Jauncey M, Micallef JM, Gilmour S, Amin J, White PA, Rawlinson W, Kaldor JM, van Beek I, Dore GJ (2004). Clearance of hepatitis C virus after newly acquired infection in injection drug users. J Infect Dis.

[45] Paradowska-Gorycka A, Stypinska B, Pawlik A, Romanowska-Prochnicka K, Haladyj E, Manczak M, Olesinska M (2016). RORC2 genetic variants and serum levels in patients with rheumatoid arthritis. Int J Mol Sci.

