# Supplementary material for: Polymorphisms in the Th17 cell-related RORC gene are associated with spontaneous clearance of HCV in Chinese women
Source: BMC Infect Dis. 2018 Jun 4;18:254. doi: 10.1186/s12879-018-3153-2 (PMC5987631; doi:10.1186/s12879-018-3153-2)
Supplement: Supplementary file 1 — Figure S1. Comparison of distributions of IFNL3 and RORC SNP genotypes among HIVneg, HIVpos, and total resolved individuals. (a) Distribution of genotypes (%) of three IFNL3 SNPs (rs12979860, rs8099917, and rs12980275). (b) Distribution of genotypes (%) of two RORC SNPs (rs9826, and rs1521177). Chi-square (χ2) and Fisher’s exact tests were used to evaluate the differences in SNP distributions between two groups. P-values (two-tailed) < 0.05 were considered significant (n.s., not significant). Figure S2. HCV viral load levels of RORC SNP genotypes (rs9826, rs1521177) among HIVneg HCV carriers of the primary cohort. Figure S3. Linkage disequilibrium tests for RORC SNPs (rs9826 and rs1521177) and IFNL3 SNPs (rs12979860, rs8099917 and rs12980275) in the primary cohort (a) D’ value. (b) r2 value. Table S1. Allele frequencies of IFNL3 gene SNPs in different populations. Table S2. Allele, genotype, and carrier frequencies and percentages of tested SNPs in the primary cohort. Table S3. RORC gene SNP allele frequencies in different populations. Table S4. The Hardy–Weinberg Equilibrium (HWE) test of all SNPs in the whole study population. Table S5. Genotype and allele frequency distributions of RORC SNPs in HIVpos group. Table S6. Genotype and allele frequency distributions of RORC SNPs in HIVneg HCV carriers of the primary cohort. Table S7. Linkage disequilibrium tests for RORC rs9826/rs1521177 in the primary cohort and the IFNL3 favorable sub-cohort. (DOC 1671 kb) [file 12879_2018_3153_MOESM1_ESM.doc]

**Supplementary material (Supplementary Figure S1-S3 and Tables S1–S7)** for **“****Polymorphisms in the Th17 cell-related *RORC* gene are associated with spontaneous clearance of HCV in Chinese women”**

**Figure S1**

**
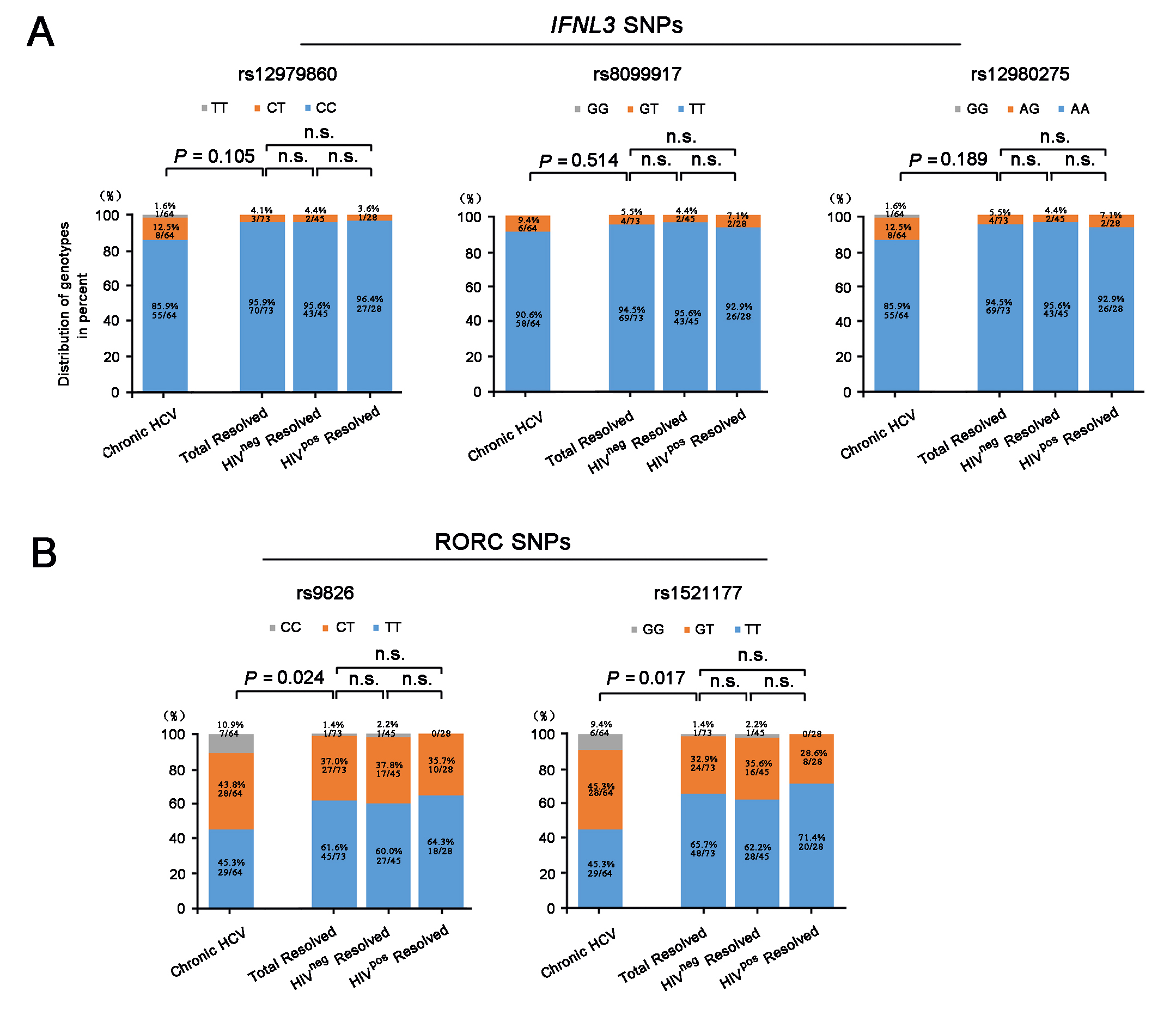
**

**Figure S1. Comparison of distributions of *IFNL3* and *RORC* SNP genotypes among HIV^neg^, HIV^pos^, and total resolved individuals. (A)** Distribution of genotypes (%) of three *IFNL3* SNPs (rs12979860, rs8099917, and rs12980275). **(B)** Distribution of genotypes (%) of two *RORC* SNPs ( rs9826, and rs1521177). Chi-square (χ^2^) and Fisher’s exact tests were used to evaluate the differences in SNP distributions between two groups. *P*-values (two-tailed) <0.05 were considered significant (n.s., not significant).

**Figure S2**


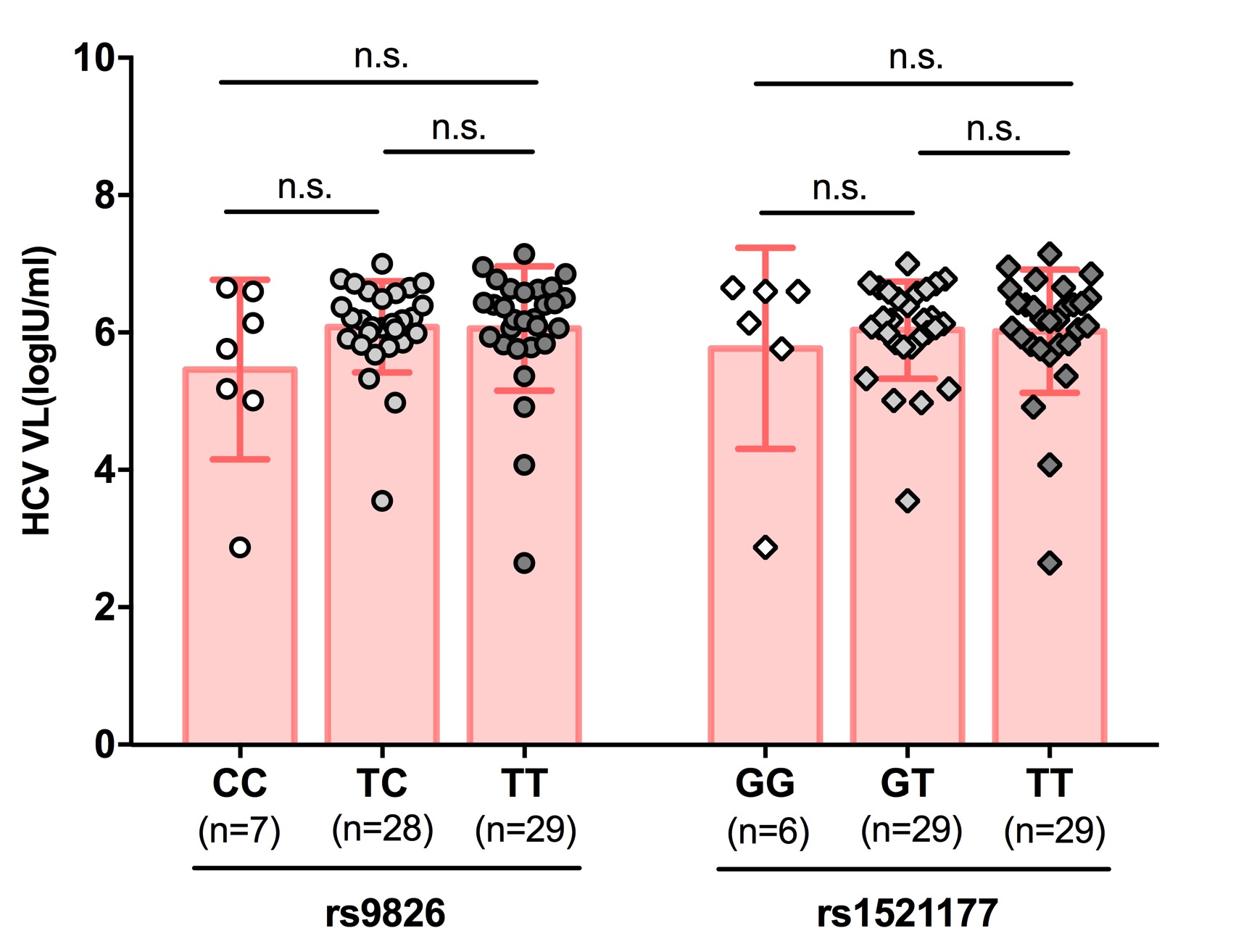


**Figure S2. HCV viral load levels of *RORC* SNP genotypes (rs9826, rs1521177) among HIV^neg^ HCV carriers of the primary cohort.**

**Figure S3**

**
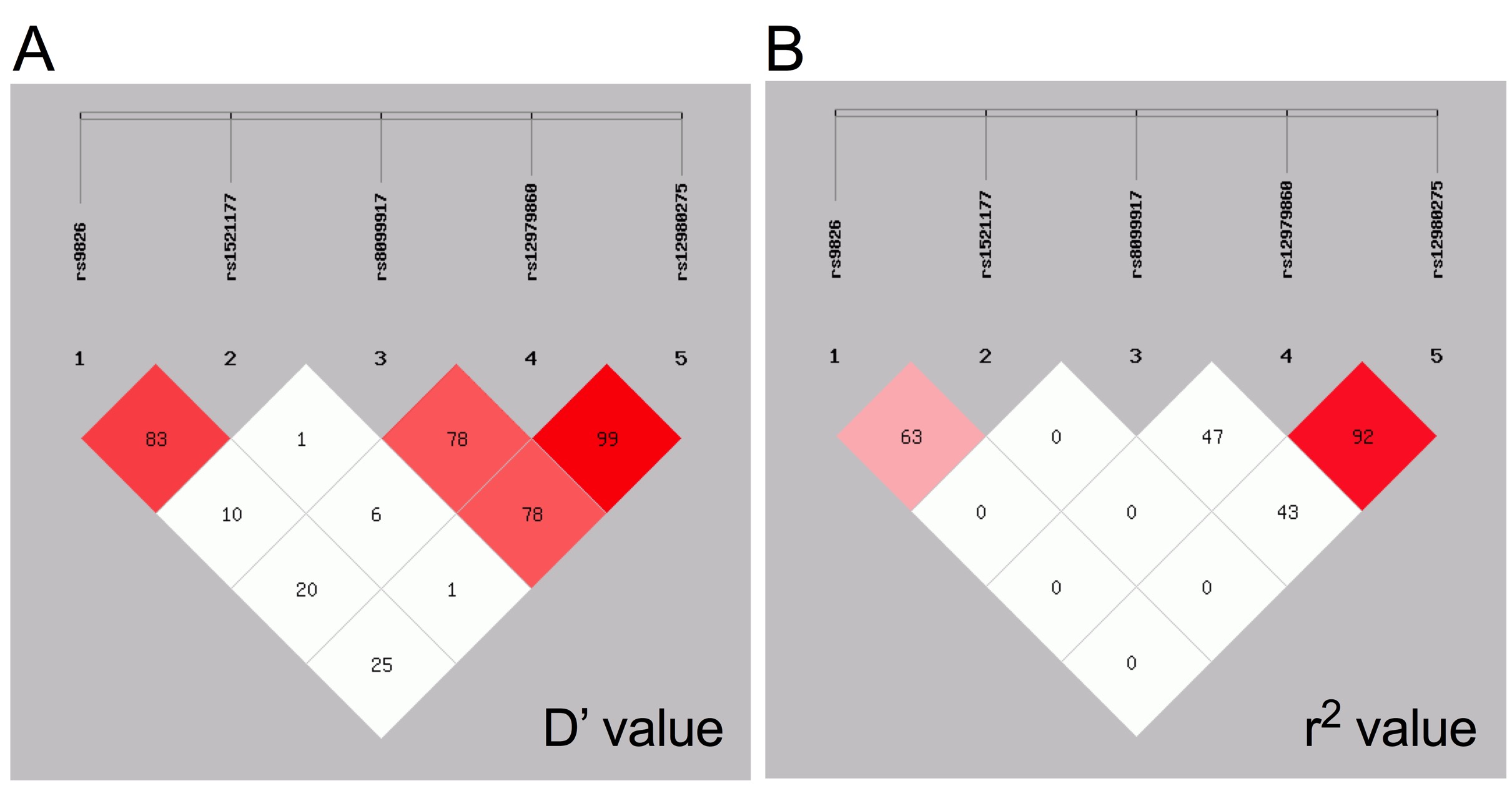
**

**Figure S3. Linkage disequilibrium tests for RORC SNPs (rs9826 and rs1521177) and IFNL3 SNPs (rs12979860, rs8099917 and rs12980275) in the primary cohort (A)** D’ value. **(B)** r^2^ value.

**Table S1.** Allele frequencies of *IFNL3* gene SNPs in different populations

|  | **rs12979860** | | | **rs8099917** | | | **rs12980275** | | |
| --- | --- | --- | --- | --- | --- | --- | --- | --- | --- |
| **Population** | Allele frequencies | Allele frequencies | Total count | Allele frequencies | Allele frequencies | Total count | Allele frequencies | Allele frequencies | Total count |
|  | C | T |  | T | G |  | A | G |  |
| ACB | 0.2865 | 0.7135 | 192 | 0.2865 | 0.7135 | 192 | 0.3319 | 0.6681 | 232 |
| ASW | 0.3197 | 0.6803 | 122 | 0.3197 | 0.6803 | 122 | 0.4344 | 0.5656 | 122 |
| BEB | 0.8023 | 0.1977 | 172 | 0.8023 | 0.1977 | 172 | 0.8314 | 0.1686 | 172 |
| CEU | 0.7273 | 0.2727 | 198 | 0.7273 | 0.2727 | 198 | 0.7323 | 0.2677 | 198 |
| CLM | 0.5851 | 0.4149 | 188 | 0.5851 | 0.4149 | 188 | 0.5745 | 0.4255 | 188 |
| ESN | 0.2879 | 0.7121 | 198 | 0.2879 | 0.7121 | 198 | 0.3788 | 0.6212 | 198 |
| FIN | 0.7323 | 0.2677 | 198 | 0.7323 | 0.2677 | 198 | 0.7374 | 0.2626 | 198 |
| GBR | 0.6923 | 0.3077 | 182 | 0.6923 | 0.3077 | 182 | 0.7198 | 0.2802 | 182 |
| GIH | 0.7621 | 0.2379 | 206 | 0.7621 | 0.2379 | 206 | 0.7961 | 0.2039 | 206 |
| GWD | 0.2699 | 0.7301 | 226 | 0.2699 | 0.7301 | 226 | 0.4115 | 0.5885 | 226 |
| IBS | 0.7056 | 0.2944 | 214 | 0.7056 | 0.2944 | 214 | 0.7009 | 0.2991 | 214 |
| ITU | 0.7647 | 0.2353 | 204 | 0.7647 | 0.2353 | 204 | 0.7598 | 0.2402 | 204 |
| JPT | 0.8990 | 0.1010 | 208 | 0.8990 | 0.1010 | 208 | 0.8846 | 0.1154 | 208 |
| KHV | 0.9091 | 0.0909 | 198 | 0.9091 | 0.0909 | 198 | 0.8939 | 0.1061 | 198 |
| LWK | 0.4848 | 0.5152 | 198 | 0.4848 | 0.5152 | 198 | 0.5556 | 0.4444 | 198 |
| MSL | 0.3471 | 0.6529 | 170 | 0.3471 | 0.6529 | 170 | 0.5000 | 0.5000 | 170 |
| MXL | 0.5391 | 0.4609 | 128 | 0.5391 | 0.4609 | 128 | 0.5820 | 0.4180 | 122 |
| PEL | 0.6235 | 0.3765 | 170 | 0.6235 | 0.3765 | 170 | 0.6118 | 0.3882 | 170 |
| PJL | 0.7604 | 0.2396 | 192 | 0.7604 | 0.2396 | 192 | 0.7656 | 0.2344 | 192 |
| PUR | 0.6346 | 0.3654 | 208 | 0.6346 | 0.3654 | 208 | 0.6538 | 0.3462 | 208 |
| STU | 0.7500 | 0.2500 | 204 | 0.7500 | 0.2500 | 204 | 0.7598 | 0.2402 | 204 |
| TSI | 0.6028 | 0.3972 | 214 | 0.6028 | 0.3972 | 214 | 0.6028 | 0.3972 | 214 |
| YRI | 0.3241 | 0.6759 | 216 | 0.3241 | 0.6759 | 216 | 0.4352 | 0.5648 | 216 |
| CDX | 0.8978 | 0.1022 | 186 | 0.8978 | 0.1022 | 186 | 0.8978 | 0.1022 | 186 |
| CHB | 0.9369 | 0.0631 | 206 | 0.9369 | 0.0631 | 206 | 0.9320 | 0.0680 | 206 |
| CHS | 0.9524 | 0.0476 | 210 | 0.9524 | 0.0476 | 210 | 0.9429 | 0.0571 | 210 |
| **Primary cohort** | 0.9560 | 0.0439 | 274 | 0.9621 | 0.0379 | 274 | 0.9489 | 0.0511 | 274 |

*P*-values (χ^2^ test) for comparison of allele distributions in the primary cohort and CDX, CHB, and CHS populations were as follows: rs12979860, cohort vs. CHB (0.408), CHS (0.830), and CDX (0.014); rs8099917, cohort vs. CHB (0.199), CHS (0.646), and CDX (0.006); and rs12980275, cohort vs. CHB (0.439), CHS (0.840), and CDX (0.043). There were no significant differences in allele distribution between the study cohort and CHB or CHS populations, while there was a significant difference between the study cohort and CDX.

All data are from <https://www.ncbi.nlm.nih.gov/variation/tools/1000genomes/> EXCEPT the study cohort. ACB, African Caribbeans in Barbados; ASW, Americans of African Ancestry in SW USA; BEB, Bengali from Bangladesh; CDX, Chinese Dai in Xishuangbanna, China; CEU, Utah Residents (CEPH) with Northern and Western Ancestry; CHB, Han Chinese in Beijing, China; CHS, Southern Han Chinese; CLM, Colombians from Medellin, Colombia; ESN, Esan in Nigeria; FIN, Finnish in Finland; GBR, British in England and Scotland; GIH, Gujarati Indian from Houston, Texas; GWD, Gambian in Western Divisions in the Gambia; IBS, Iberian Population in Spain; ITU, Indian Telugu from the UK; JPT, Japanese in Tokyo, Japan; KHV, Kinh in Ho Chi Minh City, Vietnam; LWK, Luhya in Webuye, Kenya; MSL, Mende in Sierra Leone; MXL, Mexican Ancestry from Los Angeles USA; PEL, Peruvians from Lima, Peru; PJL, Punjabi from Lahore, Pakistan; PUR, Puerto Ricans from Puerto Rico; STU, Sri Lankan Tamil from the UK; TSI, Toscani in Italia; YRI, Yoruba in Ibadan, Nigeria.

**Table S2.** Allele, genotype, and carrier frequencies and percentages of tested SNPs in the primary cohort

|  |  | | **Chronic group** | | **Resolved group** | |  |  |
| --- | --- | --- | --- | --- | --- | --- | --- | --- |
| SNP | Genotype | Number | | Frequency | Number | Frequency | *P*-value | Function |
| ***IRF3* gene (chromosome 19)** | | | | | | | | |
| rs7251 | CC | 22 | | 0.349 | 26 | 0.366 | 0.8413 | missense |
|  | GG | 8 | | 0.127 | 11 | 0.155 |  |  |
|  | CG | 33 | | 0.524 | 34 | 0.479 |  |  |
|  | C allele | 77 | | 0.611 | 86 | 0.606 | 1 |  |
|  | G allele | 49 | | 0.389 | 56 | 0.394 |  |  |
| rs2304206 | AA | 1 | | 0.019 | 1 | 0.015 | 0.894 | intron variant |
|  | GG | 45 | | 0.833 | 54 | 0.806 |  |  |
|  | AG | 8 | | 0.148 | 12 | 0.179 |  |  |
|  | A allele | 10 | | 0.093 | 14 | 0.104 | 0.8309 |  |
|  | G allele | 98 | | 0.907 | 120 | 0.896 |  |  |
| rs2304205 | AA | 49 | | 0.766 | 52 | 0.712 | 0.744 | 5′ UTR |
|  | CC | 1 | | 0.016 | 2 | 0.027 |  |  |
|  | AC | 14 | | 0.218 | 19 | 0.260 |  |  |
|  | A allele | 112 | | 0.875 | 123 | 0.842 | 0.491 |  |
|  | C allele | 16 | | 0.125 | 23 | 0.158 |  |  |
| rs2304204 | CC | 4 | | 0.063 | 5 | 0.070 | 0.5197 | 5′ UTR |
|  | TT | 39 | | 0.609 | 49 | 0.690 |  |  |
|  | CT | 21 | | 0.328 | 17 | 0.239 |  |  |
|  | C allele | 29 | | 0.227 | 27 | 0.190 | 0.5480 |  |
|  | T allele | 99 | | 0.773 | 115 | 0.810 |  |  |
| ***IFNG* (chromosome 12)** | | | | | | | | |
| rs2069718 | AA | 50 | | 0.794 | 56 | 0.789 | 0.9443 | intron variant |
|  | GG | 0 | | 0.000 | 0 | 0.000 |  |  |
|  | AG | 13 | | 0.206 | 15 | 0.211 |  |  |
|  | A allele | 113 | | 0.897 | 127 | 0.894 | 1 |  |
|  | G allele | 13 | | 0.103 | 15 | 0.106 |  |  |
| rs1861494 | CC | 12 | | 0.188 | 12 | 0.171 | 0.9601 | intron variant |
|  | TT | 19 | | 0.297 | 22 | 0.314 |  |  |
|  | CT | 33 | | 0.516 | 36 | 0.514 |  |  |
|  | C allele | 57 | | 0.445 | 60 | 0.429 | 0.8061 |  |
|  | T allele | 71 | | 0.555 | 80 | 0.571 |  |  |
| rs2069727 | TT | 51 | | 0.823 | 56 | 0.800 | 0.8256 | downstream variant (500 bp) |
|  | CC | 0 | | 0.000 | 0 | 0.000 |  |  |
|  | CT | 11 | | 0.177 | 14 | 0.200 |  |  |
|  | T allele | 113 | | 0.911 | 126 | 0.900 | 0.8348 |  |
|  | C allele | 11 | | 0.089 | 14 | 0.100 |  |  |
| rs2430561 | AA | 0 | | 0.000 | 0 | 0.000 | 0.8260 | intron variant |
|  | TT | 53 | | 0.828 | 58 | 0.806 |  |  |
|  | AT | 11 | | 0.172 | 14 | 0.194 |  |  |
|  | A allele | 11 | | 0.086 | 14 | 0.097 | 0.7478 |  |
|  | T allele | 117 | | 0.914 | 130 | 0.903 |  |  |
| ***IL4* (chromosome 5)** | | | | | | | | |
| rs2243248 | GG | 0 | | 0.000 | 0 | 0.000 | 0.7549 | upstream variant (2 kb) |
|  | TT | 56 | | 0.903 | 65 | 0.929 |  |  |
|  | GT | 6 | | 0.097 | 5 | 0.071 |  |  |
|  | G allele | 6 | | 0.048 | 5 | 0.036 | 0.7601 |  |
|  | T allele | 118 | | 0.952 | 135 | 0.964 |  |  |
| rs2243250 | TT | 42 | | 0.656 | 47 | 0.662 | 0.5214 | upstream variant (3 kb) |
|  | CC | 3 | | 0.047 | 1 | 0.014 |  |  |
|  | CT | 19 | | 0.297 | 23 | 0.324 |  |  |
|  | T allele | 103 | | 0.805 | 117 | 0.824 | 0.6842 |  |
|  | C allele | 25 | | 0.195 | 25 | 0.176 |  |  |
| rs2070874 | TT | 42 | | 0.656 | 46 | 0.657 | 0.5229 | 5′ UTR |
|  | CC | 3 | | 0.047 | 1 | 0.014 |  |  |
|  | CT | 19 | | 0.297 | 23 | 0.329 |  |  |
|  | T allele | 103 | | 0.805 | 115 | 0.821 | 0.7554 |  |
|  | C allele | 25 | | 0.195 | 25 | 0.179 |  |  |
| rs2243268 | AA | 3 | | 0.048 | 1 | 0.014 | 0.5211 | intron variant |
|  | CC | 41 | | 0.651 | 46 | 0.657 |  |  |
|  | AC | 19 | | 0.302 | 23 | 0.329 |  |  |
|  | A allele | 25 | | 0.198 | 25 | 0.179 | 0.7539 |  |
|  | C allele | 101 | | 0.802 | 115 | 0.821 |  |  |
| rs2227284 | GG | 2 | | 0.031 | 1 | 0.014 | 0.7258 | intron variant |
|  | TT | 49 | | 0.766 | 54 | 0.750 |  |  |
|  | GT | 13 | | 0.203 | 17 | 0.236 |  |  |
|  | G allele | 17 | | 0.133 | 19 | 0.132 | 1 |  |
|  | T allele | 111 | | 0.867 | 125 | 0.868 |  |  |
| ***TBX21* (chromosome 17)** | | | | | | | | |
| rs4794067 | TT | 47 | | 0.734 | 55 | 0.753 | 0.8306 | upstream variant (2 kb) |
|  | CC | 1 | | 0.016 | 2 | 0.027 |  |  |
|  | CT | 16 | | 0.250 | 16 | 0.219 |  |  |
|  | T allele | 110 | | 0.859 | 126 | 0.863 | 1 |  |
|  | C allele | 18 | | 0.141 | 20 | 0.137 |  |  |
| rs17250932 | TT | 50 | | 0.781 | 62 | 0.849 | 0.3034 | upstream variant (2 kb) |
|  | CC | 0 | | 0.000 | 0 | 0.000 |  |  |
|  | CT | 14 | | 0.219 | 11 | 0.151 |  |  |
|  | T allele | 114 | | 0.891 | 135 | 0.925 | 0.4018 |  |
|  | C allele | 14 | | 0.109 | 11 | 0.075 |  |  |
| rs10514934 | TT | 47 | | 0.734 | 52 | 0.712 | 0.8812 | intron variant |
|  | CC | 1 | | 0.016 | 2 | 0.027 |  |  |
|  | CT | 16 | | 0.250 | 19 | 0.260 |  |  |
|  | T allele | 110 | | 0.859 | 123 | 0.842 | 0.7366 |  |
|  | C allele | 18 | | 0.141 | 23 | 0.158 |  |  |
| ***IFNL3* (chromosome 19)** | | | | | | | | |
| rs11882871 | AA | 55 | | 0.873 | 69 | 0.958 | 0.1127 | 3′ UTR |
|  | GG | 0 | | 0.000 | 0 | 0.000 |  |  |
|  | AG | 8 | | 0.127 | 3 | 0.042 |  |  |
|  | A allele | 118 | | 0.937 | 141 | 0.979 | 0.1205 |  |
|  | G allele | 8 | | 0.063 | 3 | 0.021 |  |  |
| rs11881222 | AA | 57 | | 0.905 | 69 | 0.958 | 0.3035 | intron variant |
|  | GG | 0 | | 0.000 | 0 | 0.000 |  |  |
|  | AG | 6 | | 0.095 | 3 | 0.042 |  |  |
|  | A allele | 120 | | 0.952 | 141 | 0.979 | 0.3119 |  |
|  | G allele | 6 | | 0.048 | 3 | 0.021 |  |  |
| rs4803219 | CC | 61 | |  | 73 |  |  | 5′ UTR |
| rs12980275 | AA | 55 | | 0.859 | 69 | 0.959 | 0.1885 | near the *IFNL3* gene |
|  | GG | 1 | | 0.016 | 0 | 0.000 |  |  |
|  | AG | 8 | | 0.125 | 4 | 0.041 |  |  |
|  | A allele | 118 | | 0.921 | 142 | 0.973 | 0.0959 |  |
|  | G allele | 10 | | 0.078 | 4 | 0.027 |  |  |
| rs8099917 | GG | 0 | | 0.000 | 0 | 0.000 | 0.5144 | near the *IFNL3* gene |
|  | TT | 58 | | 0.906 | 69 | 0.959 |  |  |
|  | GT | 6 | | 0.094 | 4 | 0.041 |  |  |
|  | G allele | 6 | | 0.047 | 4 | 0.027 | 0.5225 |  |
|  | T allele | 122 | | 0.953 | 142 | 0.973 |  |  |
| rs12979860 | CC | 55 | | 0.859 | 70 | 0.959 | 0.1054 | near the *IFNL3* gene |
|  | TT | 1 | | 0.016 | 0 | 0.000 |  |  |
|  | TC | 8 | | 0.125 | 3 | 0.041 |  |  |
|  | C allele | 118 | | 0.922 | 143 | 0.979 | 0.0425 |  |
|  | T allele | 10 | | 0.078 | 3 | 0.021 |  |  |
| ***FOXP3* (chromosome X)** | | | | | | | | |
| rs2280883 | CC | 4 | | 0.063 | 5 | 0.069 | 0.7896 | intron variant |
|  | TT | 41 | | 0.641 | 42 | 0.583 |  |  |
|  | CT | 19 | | 0.297 | 25 | 0.347 |  |  |
|  | C allele | 27 | | 0.211 | 35 | 0.243 | 0.5646 |  |
|  | T allele | 101 | | 0.789 | 109 | 0.757 |  |  |
| rs3761548 | GG | 38 | | 0.594 | 42 | 0.592 | 0.6573 | intron variant |
|  | TT | 3 | | 0.047 | 6 | 0.085 |  |  |
|  | GT | 23 | | 0.359 | 23 | 0.324 |  |  |
|  | G allele | 99 | | 0.773 | 107 | 0.754 | 0.7748 |  |
|  | T allele | 29 | | 0.227 | 35 | 0.246 |  |  |
| ***RORC* (chromosome 1)** | | | | | | | | |
| rs9826 | CC | 7 | | 0.109 | 1 | 0.014 | **0.0244** | 3′ UTR |
|  | TT | 29 | | 0.453 | 45 | 0.616 |  |  |
|  | CT | 28 | | 0.438 | 27 | 0.370 |  |  |
|  | C allele | 42 | | 0.328 | 29 | 0.199 | **0.0147** |  |
|  | T allele | 86 | | 0.672 | 117 | 0.801 |  |  |
| rs10494269 | CC | 39 | | 0.609 | 51 | 0.699 | 0.4869 | intron variant |
|  | GG | 2 | | 0.031 | 1 | 0.014 |  |  |
|  | CG | 23 | | 0.359 | 21 | 0.288 |  |  |
|  | C allele | 101 | | 0.789 | 123 | 0.842 | 0.2535 |  |
|  | G allele | 27 | | 0.211 | 23 | 0.158 |  |  |
| rs1521177 | GG | 6 | | 0.094 | 1 | 0.014 | **0.0168** | intron variant |
|  | TT | 29 | | 0.453 | 48 | 0.658 |  |  |
|  | GT | 29 | | 0.453 | 24 | 0.329 |  |  |
|  | G allele | 41 | | 0.320 | 26 | 0.178 | **0.0063** |  |
|  | T allele | 87 | | 0.680 | 120 | 0.822 |  |  |
| rs7540530 | AA | 7 | | 0.109 | 14 | 0.192 | 0.3983 | intron variant |
|  | GG | 36 | | 0.563 | 36 | 0.493 |  |  |
|  | AG | 21 | | 0.328 | 23 | 0.315 |  |  |
|  | A allele | 35 | | 0.273 | 51 | 0.349 | 0.1769 |  |
|  | G allele | 93 | | 0.727 | 95 | 0.651 |  |  |
| ***TLR3* (chromosome 4)** | | | | | | | | |
| rs5743303 | AA | 45 | | 0.763 | 55 | 0.786 | 0.3474 | upstream variant |
|  | TT | 0 | | 0.000 | 2 | 0.029 |  |  |
|  | AT | 14 | | 0.237 | 13 | 0.186 |  |  |
|  | A allele | 104 | | 0.881 | 123 | 0.879 | 1 |  |
|  | T allele | 14 | | 0.119 | 17 | 0.121 |  |  |
| rs5743305 | AA | 4 | | 0.063 | 5 | 0.068 | 0.7650 | upstream variant (3 kb) |
|  | TT | 35 | | 0.556 | 36 | 0.493 |  |  |
|  | AT | 24 | | 0.381 | 32 | 0.438 |  |  |
|  | A allele | 32 | | 0.254 | 42 | 0.288 | 0.5857 |  |
|  | T allele | 94 | | 0.746 | 104 | 0.712 |  |  |
| rs11721827 | AA | 39 | | 0.609 | 38 | 0.528 | 0.8690 | intron variant |
|  | CC | 3 | | 0.047 | 3 | 0.042 |  |  |
|  | AC | 22 | | 0.344 | 31 | 0.431 |  |  |
|  | A allele | 100 | | 0.781 | 107 | 0.743 | 0.4798 |  |
|  | C allele | 28 | | 0.219 | 37 | 0.257 |  |  |
| rs13126816 | AA | 1 | | 0.016 | 4 | 0.056 | 0.4447 | intron variant |
|  | GG | 46 | | 0.719 | 48 | 0.667 |  |  |
|  | AG | 17 | | 0.266 | 20 | 0.278 |  |  |
|  | A allele | 19 | | 0.148 | 28 | 0.194 | 0.339 |  |
|  | G allele | 109 | | 0.852 | 116 | 0.806 |  |  |
| rs3775296 | AA | 5 | | 0.078 | 3 | 0.042 | 0.4523 | 5′ UTR |
|  | CC | 34 | | 0.531 | 45 | 0.625 |  |  |
|  | AC | 25 | | 0.391 | 24 | 0.333 |  |  |
|  | A allele | 35 | | 0.273 | 30 | 0.208 | 0.2545 |  |
|  | C allele | 93 | | 0.727 | 114 | 0.792 |  |  |
| rs7668666 | AA | 6 | | 0.097 | 4 | 0.057 | 0.4764 | intron variant |
|  | CC | 29 | | 0.468 | 29 | 0.414 |  |  |
|  | AC | 27 | | 0.435 | 37 | 0.529 |  |  |
|  | A allele | 39 | | 0.315 | 45 | 0.321 | 1 |  |
|  | C allele | 85 | | 0.685 | 95 | 0.679 |  |  |
| rs3775291 | TT | 6 | | 0.094 | 8 | 0.110 | 0.7101 | missense |
|  | CC | 29 | | 0.453 | 37 | 0.507 |  |  |
|  | CT | 29 | | 0.453 | 28 | 0.384 |  |  |
|  | T allele | 41 | | 0.320 | 44 | 0.301 | 0.7939 |  |
|  | C allele | 87 | | 0.680 | 102 | 0.699 |  |  |
| ***TLR7* (chromosome X)** | | | | | | | | |
| rs5935436 | TT | 0 | | 0.000 | 0 | 0.000 | 0.5963 | upstream variant (2 kb) |
|  | CC | 58 | | 0.906 | 63 | 0.875 |  |  |
|  | CT | 6 | | 0.094 | 9 | 0.125 |  |  |
|  | T allele | 6 | | 0.047 | 9 | 0.063 | 0.6069 |  |
|  | C allele | 122 | | 0.953 | 135 | 0.938 |  |  |
| rs179012 | AA | 2 | | 0.031 | 1 | 0.014 | 0.4386 | intron variant |
|  | GG | 52 | | 0.813 | 55 | 0.753 |  |  |
|  | AG | 10 | | 0.156 | 17 | 0.233 |  |  |
|  | A allele | 14 | | 0.109 | 19 | 0.130 | 0.7106 |  |
|  | G allele | 114 | | 0.891 | 127 | 0.870 |  |  |
| rs1634319 | TT | 58 | | 0.906 | 64 | 0.889 | 0.7847 | intron variant |
|  | CC | 0 | | 0.000 | 0 | 0.000 |  |  |
|  | CT | 6 | | 0.094 | 8 | 0.111 |  |  |
|  | T allele | 122 | | 0.953 | 136 | 0.944 | 0.7903 |  |
|  | C allele | 6 | | 0.047 | 8 | 0.056 |  |  |
| rs179008 | AA | 64 | |  | 73 |  |  | missense |
| rs3853839 | CC | 6 | | 0.094 | 2 | 0.029 | 0.2819 | 3′ UTR |
|  | GG | 33 | | 0.516 | 39 | 0.557 |  |  |
|  | CG | 25 | | 0.391 | 29 | 0.414 |  |  |
|  | C allele | 37 | | 0.289 | 33 | 0.236 | 0.3333 |  |
|  | G allele | 91 | | 0.711 | 107 | 0.764 |  |  |
| rs1634323 | AA | 56 | | 0.875 | 69 | 0.945 | 0.2254 | intron variant |
|  | GG | 0 | | 0.000 | 0 | 0.000 |  |  |
|  | AG | 8 | | 0.125 | 4 | 0.055 |  |  |
|  | A allele | 120 | | 0.938 | 142 | 0.973 | 0.2363 |  |
|  | G allele | 8 | | 0.063 | 4 | 0.027 |  |  |
| rs179016 | CC | 4 | | 0.063 | 1 | 0.014 | 0.1731 | intron variant |
|  | GG | 46 | | 0.719 | 49 | 0.671 |  |  |
|  | CG | 14 | | 0.219 | 23 | 0.315 |  |  |
|  | C allele | 22 | | 0.172 | 25 | 0.171 | 1 |  |
|  | G allele | 106 | | 0.828 | 121 | 0.829 |  |  |
| rs179009 | AA | 47 | | 0.734 | 46 | 0.639 | 0.1747 | intron variant |
|  | GG | 4 | | 0.063 | 2 | 0.028 |  |  |
|  | AG | 13 | | 0.203 | 24 | 0.333 |  |  |
|  | A allele | 107 | | 0.836 | 116 | 0.806 | 0.5318 |  |
|  | G allele | 21 | | 0.164 | 28 | 0.194 |  |  |
| ***TLR9* (chromosome 3)** | | | | | | | | |
| rs352140 | TT | 7 | | 0.113 | 10 | 0.137 | 0.5686 | synonymous codon |
|  | CC | 23 | | 0.371 | 32 | 0.438 |  |  |
|  | CT | 32 | | 0.516 | 31 | 0.425 |  |  |
|  | T allele | 46 | | 0.371 | 51 | 0.349 | 0.7992 |  |
|  | C allele | 78 | | 0.629 | 95 | 0.651 |  |  |
| rs352139 | CC | 7 | | 0.111 | 14 | 0.203 | 0.2855 | intron variant |
|  | TT | 22 | | 0.349 | 25 | 0.362 |  |  |
|  | CT | 34 | | 0.540 | 30 | 0.435 |  |  |
|  | C allele | 48 | | 0.381 | 58 | 0.420 | 0.532 |  |
|  | T allele | 78 | | 0.619 | 80 | 0.580 |  |  |
| rs5743836 | A | 63 | |  | 72 |  |  | upstream variant (2 kb) |
| rs187084 | AA | 22 | | 0.355 | 32 | 0.438 | 0.4470 | upstream variant (3 kb) |
|  | GG | 9 | | 0.113 | 11 | 0.151 |  |  |
|  | AG | 33 | | 0.532 | 30 | 0.411 |  |  |
|  | A allele | 77 | | 0.602 | 94 | 0.653 | 0.4710 |  |
|  | G allele | 51 | | 0.398 | 52 | 0.347 |  |  |
| ***CXCR5* (chromosome 11)** | | | | | | | | |
| rs1623316 | CC | 9 | | 0.148 | 14 | 0.203 | 0.5435 | intron variant |
|  | GG | 20 | | 0.328 | 25 | 0.362 |  |  |
|  | CG | 32 | | 0.525 | 30 | 0.435 |  |  |
|  | C allele | 50 | | 0.410 | 58 | 0.420 | 0.9 |  |
|  | G allele | 72 | | 0.590 | 80 | 0.580 |  |  |
| rs3922 | AA | 34 | | 0.557 | 36 | 0.529 | 0.9471 | 3′ UTR |
|  | GG | 4 | | 0.066 | 5 | 0.074 |  |  |
|  | AG | 23 | | 0.377 | 27 | 0.397 |  |  |
|  | A allele | 91 | | 0.746 | 99 | 0.728 | 0.7784 |  |
|  | G allele | 31 | | 0.254 | 37 | 0.272 |  |  |
| rs497916 | TT | 2 | | 0.031 | 3 | 0.042 | 0.7948 | intron variant |
|  | CC | 44 | | 0.688 | 45 | 0.634 |  |  |
|  | CT | 18 | | 0.281 | 23 | 0.324 |  |  |
|  | T allele | 22 | | 0.172 | 29 | 0.204 | 0.536 |  |
|  | C allele | 106 | | 0.828 | 113 | 0.796 |  |  |
| rs676925 | CC | 47 | | 0.839 | 59 | 0.855 | 0.6012 | 3′ UTR |
|  | GG | 0 | | 0.000 | 1 | 0.014 |  |  |
|  | CG | 9 | | 0.161 | 9 | 0.130 |  |  |
|  | C allele | 103 | | 0.920 | 127 | 0.920 | 1 |  |
|  | G allele | 9 | | 0.080 | 11 | 0.080 |  |  |
| ***ICOS* (chromosome 2)** | | | | | | | | |
| rs10183087 | AA | 51 | | 0.823 | 58 | 0.817 | 1 | 3′ UTR |
|  | CC | 0 | | 0.000 | 0 | 0.000 |  |  |
|  | AC | 11 | | 0.177 | 13 | 0.183 |  |  |
|  | A allele | 113 | | 0.911 | 129 | 0.908 | 1 |  |
|  | C allele | 11 | | 0.089 | 13 | 0.092 |  |  |
| rs4404254 | TT | 51 | | 0.810 | 59 | 0.819 | 1 | 3′ UTR |
|  | CC | 0 | | 0.000 | 0 | 0.000 |  |  |
|  | CT | 12 | | 0.190 | 13 | 0.181 |  |  |
|  | T allele | 114 | | 0.905 | 131 | 0.910 | 1 |  |
|  | C allele | 12 | | 0.095 | 13 | 0.090 |  |  |
| rs4452124 | TT | 30 | | 0.469 | 29 | 0.403 | 0.1928 | intron variant |
|  | CC | 2 | | 0.031 | 8 | 0.111 |  |  |
|  | CT | 32 | | 0.500 | 35 | 0.486 |  |  |
|  | T allele | 92 | | 0.719 | 93 | 0.646 | 0.2412 |  |
|  | C allele | 36 | | 0.281 | 51 | 0.354 |  |  |
| rs11571323 | AA | 0 | | 0.000 | 0 | 0.000 | 1 | intron variant |
|  | GG | 56 | | 0.903 | 65 | 0.915 |  |  |
|  | AG | 6 | | 0.097 | 6 | 0.085 |  |  |
|  | A allele | 6 | | 0.048 | 6 | 0.042 | 1 |  |
|  | G allele | 118 | | 0.952 | 136 | 0.958 |  |  |
| rs4335928 | TT | 46 | | 0.719 | 51 | 0.699 | 0.4331 | intron variant |
|  | CC | 3 | | 0.047 | 1 | 0.014 |  |  |
|  | CT | 15 | | 0.234 | 21 | 0.288 |  |  |
|  | T allele | 107 | | 0.836 | 123 | 0.842 | 1 |  |
|  | C allele | 21 | | 0.164 | 23 | 0.158 |  |  |
| rs4675377 | TT | 31 | | 0.484 | 29 | 0.414 | 0.1608 | intron variant |
|  | CC | 7 | | 0.109 | 3 | 0.043 |  |  |
|  | CT | 26 | | 0.406 | 38 | 0.543 |  |  |
|  | T allele | 88 | | 0.688 | 96 | 0.686 | 1 |  |
|  | C allele | 40 | | 0.313 | 44 | 0.314 |  |  |
| ***IL21* (chromosome 4)** | | | | | | | | |
| rs907715 | TT | 15 | | 0.234 | 19 | 0.271 | 0.8574 | intron variant |
|  | CC | 18 | | 0.281 | 20 | 0.286 |  |  |
|  | CT | 31 | | 0.484 | 31 | 0.443 |  |  |
|  | T allele | 61 | | 0.477 | 69 | 0.493 | 0.8077 |  |
|  | C allele | 67 | | 0.523 | 71 | 0.507 |  |  |
| ***CD40LG* (chromosome X)** | | | | | | | | |
| rs3092923 | TT | 54 | | 0.857 | 59 | 0.808 | 0.382 | intron variant |
|  | CC | 0 | | 0.000 | 2 | 0.027 |  |  |
|  | CT | 9 | | 0.143 | 12 | 0.164 |  |  |
|  | T allele | 117 | | 0.929 | 130 | 0.890 | 0.3007 |  |
|  | C allele | 9 | | 0.071 | 16 | 0.110 |  |  |
| rs715762 | CC | 64 | |  | 73 |  |  | intron variant |
| rs1126535 | TT | 54 | | 0.857 | 60 | 0.845 | 1 | synonymous codon |
|  | CC | 0 | | 0.000 | 0 | 0.000 |  |  |
|  | CT | 9 | | 0.143 | 11 | 0.155 |  |  |
|  | T allele | 117 | | 0.929 | 131 | 0.923 | 1 |  |
|  | C allele | 9 | | 0.071 | 11 | 0.077 |  |  |
| rs3092933 | AA | 0 | | 0.000 | 2 | 0.027 | 0.4026 | intron variant (2 kb) |
|  | GG | 54 | | 0.844 | 59 | 0.808 |  |  |
|  | AG | 10 | | 0.156 | 12 | 0.164 |  |  |
|  | A allele | 10 | | 0.078 | 16 | 0.110 | 0.4146 |  |
|  | G allele | 118 | | 0.922 | 130 | 0.890 |  |  |
| ***CXCL13* (chromosome 4)** | | | | | | | | |
| rs3092945 | TT | 64 | |  | 73 |  |  | upstream variant |
| rs2866440 | GG | 45 | | 0.703 | 60 | 0.822 | 0.1661 | intron variant |
|  | TT | 1 | | 0.016 | 2 | 0.027 |  |  |
|  | GT | 18 | | 0.281 | 11 | 0.151 |  |  |
|  | G allele | 108 | | 0.844 | 131 | 0.897 | 0.2071 |  |
|  | T allele | 20 | | 0.156 | 15 | 0.103 |  |  |
| rs355687 | TT | 22 | | 0.344 | 35 | 0.493 | 0.1797 | intron variant |
|  | CC | 5 | | 0.078 | 6 | 0.085 |  |  |
|  | CT | 37 | | 0.578 | 30 | 0.423 |  |  |
|  | T allele | 81 | | 0.633 | 100 | 0.704 | 0.2438 |  |
|  | C allele | 47 | | 0.367 | 42 | 0.296 |  |  |
| rs924499 | CC | 51 | | 0.797 | 60 | 0.833 | 0.2645 | intron variant |
|  | GG | 0 | | 0.000 | 2 | 0.028 |  |  |
|  | CG | 13 | | 0.203 | 10 | 0.139 |  |  |
|  | C allele | 115 | | 0.898 | 130 | 0.903 | 1 |  |
|  | G allele | 13 | | 0.102 | 14 | 0.097 |  |  |
| ***BCL6* (chromosome 3)** | | | | | | | | |
| rs1056932 | AA | 38 | | 0.594 | 48 | 0.667 | 0.632 | intron variant |
|  | GG | 3 | | 0.047 | 2 | 0.028 |  |  |
|  | AG | 23 | | 0.359 | 22 | 0.306 |  |  |
|  | A allele | 99 | | 0.773 | 118 | 0.819 | 0.3673 |  |
|  | G allele | 29 | | 0.227 | 26 | 0.181 |  |  |
| rs1523475 | TT | 2 | | 0.031 | 1 | 0.014 | 0.245 | intron variant |
|  | CC | 49 | | 0.766 | 63 | 0.875 |  |  |
|  | CT | 13 | | 0.203 | 8 | 0.111 |  |  |
|  | T | 17 | | 0.133 | 10 | 0.069 | 0.1039 |  |
|  | C | 111 | | 0.867 | 134 | 0.931 |  |  |
| rs4686466 | AA | 2 | | 0.031 | 1 | 0.014 | 0.4354 | downstream variant |
|  | GG | 49 | | 0.766 | 62 | 0.849 |  |  |
|  | AG | 13 | | 0.203 | 10 | 0.137 |  |  |
|  | A allele | 17 | | 0.133 | 12 | 0.082 | 0.2373 |  |
|  | G allele | 111 | | 0.867 | 134 | 0.918 |  |  |
| ***CD81* (chromosome 11)** | | | | | | | | |
| rs2019938 | AA | 28 | | 0.438 | 20 | 0.290 | 0.1187 | intron variant |
|  | GG | 11 | | 0.172 | 10 | 0.145 |  |  |
|  | AG | 25 | | 0.391 | 39 | 0.565 |  |  |
|  | A allele | 81 | | 0.633 | 79 | 0.572 | 0.3198 |  |
|  | G allele | 47 | | 0.367 | 59 | 0.428 |  |  |
| rs71029143 | T | 20 | | 0.313 | 22 | 0.306 | 0.2141 | intron variant, upstream variant (2 kb) |
|  | DEL | 19 | | 0.297 | 13 | 0.181 |  |  |
|  | DEL.T | 25 | | 0.391 | 37 | 0.514 |  |  |
|  | T allele | 65 | | 0.508 | 81 | 0.563 | 0.3951 |  |
|  | DEL | 63 | | 0.492 | 63 | 0.438 |  |  |
| rs708154 | CC | 14 | | 0.219 | 15 | 0.205 | 0.2744 | intron variant, upstream variant (2 kb) |
|  | TT | 23 | | 0.359 | 18 | 0.247 |  |  |
|  | CT | 27 | | 0.422 | 40 | 0.548 |  |  |
|  | C allele | 55 | | 0.430 | 70 | 0.479 | 0.466 |  |
|  | T allele | 73 | | 0.570 | 76 | 0.521 |  |  |
| rs731909 | CC | 17 | | 0.266 | 15 | 0.208 | 0.1056 | intron variant |
|  | GG | 22 | | 0.344 | 16 | 0.222 |  |  |
|  | CG | 25 | | 0.391 | 41 | 0.569 |  |  |
|  | C allele | 59 | | 0.461 | 71 | 0.493 | 0.6278 |  |
|  | G allele | 69 | | 0.539 | 73 | 0.507 |  |  |
| rs800334 | GG | 17 | | 0.266 | 17 | 0.236 | 0.1123 | intron variant |
|  | TT | 23 | | 0.359 | 16 | 0.222 |  |  |
|  | GT | 24 | | 0.375 | 39 | 0.542 |  |  |
|  | G allele | 58 | | 0.453 | 73 | 0.507 | 0.3966 |  |
|  | T allele | 70 | | 0.547 | 71 | 0.493 |  |  |
| rs708564 | CC | 24 | | 0.375 | 16 | 0.219 | 0.0691 | intron variant |
|  | TT | 15 | | 0.234 | 15 | 0.205 |  |  |
|  | CT | 25 | | 0.391 | 42 | 0.575 |  |  |
|  | C | 73 | | 0.570 | 74 | 0.507 | 0.3318 |  |
|  | T allele | 55 | | 0.430 | 72 | 0.493 |  |  |
| rs7939051 | TT | 28 | |  | 61 |  |  | intron variant |
| rs800335 | CC | 24 | | 0.375 | 16 | 0.222 | 0.081 | intron variant |
|  | TT | 15 | | 0.234 | 15 | 0.208 |  |  |
|  | CT | 25 | | 0.391 | 41 | 0.569 |  |  |
|  | C allele | 73 | | 0.570 | 73 | 0.507 | 0.3305 |  |
|  | T allele | 55 | | 0.430 | 71 | 0.493 |  |  |
| ***LDLR* (chromosome 19)** | | | | | | | | |
| rs17248720 | CC | 60 | | 0.938 | 64 | 0.889 | 0.3762 | upstream variant (2 kb) |
|  | TT | 0 | | 0.000 | 0 | 0.000 |  |  |
|  | CT | 4 | | 0.063 | 8 | 0.111 |  |  |
|  | C allele | 124 | | 0.969 | 136 | 0.944 | 0.3872 |  |
|  | T allele | 4 | | 0.031 | 8 | 0.056 |  |  |
| rs6511721 | AA | 32 | | 0.500 | 34 | 0.472 | 0.8374 | intron variant |
|  | GG | 6 | | 0.094 | 9 | 0.125 |  |  |
|  | AG | 26 | | 0.406 | 29 | 0.403 |  |  |
|  | A allele | 90 | | 0.703 | 97 | 0.674 | 0.6944 |  |
|  | G allele | 38 | | 0.297 | 47 | 0.326 |  |  |
| rs1003723 | TT | 2 | | 0.031 | 1 | 0.014 | 0.5615 | intron variant |
|  | CC | 50 | | 0.781 | 53 | 0.736 |  |  |
|  | CT | 12 | | 0.188 | 18 | 0.250 |  |  |
|  | T allele | 16 | | 0.125 | 20 | 0.139 | 0.8581 |  |
|  | C allele | 112 | | 0.875 | 124 | 0.861 |  |  |
| rs6413504 | AA | 29 | | 0.453 | 39 | 0.534 | 0.4584 | intron variant |
|  | GG | 9 | | 0.141 | 6 | 0.082 |  |  |
|  | AG | 26 | | 0.406 | 28 | 0.384 |  |  |
|  | A allele | 84 | | 0.656 | 106 | 0.726 | 0.2381 |  |
|  | G allele | 44 | | 0.344 | 40 | 0.274 |  |  |
| rs14158 | AA | 4 | | 0.062 | 10 | 0.139 | 0.1556 | 3′ UTR |
|  | GG | 30 | | 0.469 | 24 | 0.333 |  |  |
|  | AG | 30 | | 0.469 | 38 | 0.528 |  |  |
|  | A allele | 38 | | 0.297 | 58 | 0.403 | 0.0759 |  |
|  | G allele | 90 | | 0.703 | 86 | 0.597 |  |  |
| rs1433099 | CC | 28 | | 0.438 | 41 | 0.586 | 0.211 | 3′ UTR |
|  | TT | 6 | | 0.094 | 6 | 0.086 |  |  |
|  | CT | 30 | | 0.469 | 23 | 0.329 |  |  |
|  | C allele | 86 | | 0.672 | 105 | 0.750 | 0.1776 |  |
|  | T allele | 42 | | 0.328 | 35 | 0.250 |  |  |
| rs2738446 | CC | 47 | | 0.734 | 49 | 0.671 | 0.3571 | intron variant |
|  | GG | 1 | | 0.016 | 0 | 0.000 |  |  |
|  | CG | 16 | | 0.250 | 24 | 0.329 |  |  |
|  | C allele | 110 | | 0.859 | 122 | 0.836 | 0.6177 |  |
|  | G allele | 18 | | 0.141 | 24 | 0.164 |  |  |
| rs2738466 | AA | 28 | | 0.438 | 23 | 0.315 | 0.1131 | 3′ UTR |
|  | GG | 3 | | 0.047 | 10 | 0.137 |  |  |
|  | AG | 33 | | 0.516 | 40 | 0.548 |  |  |
|  | A allele | 89 | | 0.695 | 86 | 0.589 | 0.078 |  |
|  | G allele | 39 | | 0.305 | 60 | 0.411 |  |  |
| ***SCARB1*** **(chromosome 12)** | | | | | | | | |
| rs838881 | AA | 48 | | 0.762 | 55 | 0.764 | 0.7704 | 3′ UTR |
|  | GG | 2 | | 0.032 | 1 | 0.014 |  |  |
|  | AG | 13 | | 0.206 | 16 | 0.222 |  |  |
|  | A allele | 109 | | 0.865 | 126 | 0.875 | 0.857 |  |
|  | G allele | 17 | | 0.135 | 18 | 0.125 |  |  |
| rs838884 | AA | 50 | | 0.820 | 55 | 0.821 | 0.3026 | 3′ UTR |
|  | GG | 2 | | 0.033 | 0 | 0.000 |  |  |
|  | AG | 9 | | 0.148 | 12 | 0.179 |  |  |
|  | A allele | 109 | | 0.893 | 122 | 0.910 | 0.6783 |  |
|  | G allele | 13 | | 0.107 | 12 | 0.090 |  |  |
| rs9919713 | AA | 27 | | 0.422 | 21 | 0.288 | 0.258 | intron variant |
|  | TT | 11 | | 0.172 | 16 | 0.219 |  |  |
|  | AT | 26 | | 0.406 | 36 | 0.493 |  |  |
|  | A allele | 80 | | 0.625 | 78 | 0.534 | 0.1425 |  |
|  | T allele | 48 | | 0.375 | 68 | 0.466 |  |  |
| rs5888 | AA | 6 | | 0.094 | 2 | 0.028 | 0.262 | synonymous codon |
|  | GG | 35 | | 0.547 | 43 | 0.597 |  |  |
|  | AG | 23 | | 0.359 | 27 | 0.375 |  |  |
|  | A allele | 35 | | 0.273 | 31 | 0.215 | 0.3213 |  |
|  | G allele | 93 | | 0.727 | 113 | 0.785 |  |  |
| rs3782287 | AA | 5 | | 0.078 | 6 | 0.082 | 0.2089 | intron variant |
|  | GG | 44 | | 0.688 | 40 | 0.548 |  |  |
|  | AG | 15 | | 0.234 | 27 | 0.370 |  |  |
|  | A allele | 25 | | 0.195 | 39 | 0.267 | 0.1978 |  |
|  | G allele | 103 | | 0.805 | 107 | 0.733 |  |  |
| rs11057830 | AA | 1 | | 0.016 | 2 | 0.029 | 0.6842 | intron variant |
|  | GG | 43 | | 0.683 | 51 | 0.729 |  |  |
|  | AG | 19 | | 0.302 | 17 | 0.243 |  |  |
|  | A allele | 21 | | 0.167 | 21 | 0.150 | 0.7386 |  |
|  | G allele | 105 | | 0.833 | 119 | 0.850 |  |  |
| rs10846744 | CC | 26 | | 0.481 | 31 | 0.470 | 0.9725 | intron variant |
|  | GG | 7 | | 0.130 | 8 | 0.121 |  |  |
|  | CG | 21 | | 0.389 | 27 | 0.409 |  |  |
|  | C allele | 73 | | 0.676 | 89 | 0.674 | 1 |  |
|  | G allele | 35 | | 0.324 | 43 | 0.326 |  |  |
| rs4765623 | TT | 11 | | 0.172 | 9 | 0.125 | 0.5426 | intron variant |
|  | CC | 23 | | 0.359 | 32 | 0.444 |  |  |
|  | CT | 30 | | 0.469 | 31 | 0.431 |  |  |
|  | T allele | 52 | | 0.406 | 49 | 0.340 | 0.3146 |  |
|  | C allele | 76 | | 0.594 | 95 | 0.660 |  |  |
| ***CLDN1* (chromosome 3)** | | | | | | | | |
| rs893051 | CC | 32 | | 0.500 | 38 | 0.535 | 0.7516 | intron variant |
|  | GG | 4 | | 0.063 | 6 | 0.085 |  |  |
|  | CG | 28 | | 0.438 | 27 | 0.380 |  |  |
|  | C allele | 92 | | 0.719 | 103 | 0.725 | 1 |  |
|  | G allele | 36 | | 0.281 | 39 | 0.275 |  |  |
| rs9842214 | TT | 3 | | 0.048 | 5 | 0.070 | 0.6279 | downstream variant (500 bp) |
|  | CC | 36 | | 0.571 | 35 | 0.493 |  |  |
|  | CT | 24 | | 0.381 | 31 | 0.437 |  |  |
|  | T allele | 30 | | 0.238 | 41 | 0.289 | 0.4058 |  |
|  | C allele | 96 | | 0.762 | 101 | 0.711 |  |  |
| rs12629166 | CC | 6 | | 0.094 | 5 | 0.069 | 0.5056 | intron variant |
|  | TT | 34 | | 0.531 | 33 | 0.458 |  |  |
|  | CT | 24 | | 0.375 | 34 | 0.472 |  |  |
|  | C allele | 36 | | 0.281 | 44 | 0.306 | 0.6907 |  |
|  | T allele | 92 | | 0.719 | 100 | 0.694 |  |  |
| rs3774025 | CC | 34 | | 0.618 | 31 | 0.544 | 0.0824 | intron variant |
|  | TT | 9 | | 0.164 | 19 | 0.333 |  |  |
|  | CT | 12 | | 0.218 | 7 | 0.123 |  |  |
|  | C allele | 80 | | 0.727 | 69 | 0.605 | 0.0656 |  |
|  | T allele | 30 | | 0.273 | 45 | 0.395 |  |  |
| rs6800425 | AA | 6 | | 0.094 | 8 | 0.110 | 0.2243 | intron variant |
|  | GG | 33 | | 0.516 | 27 | 0.370 |  |  |
|  | AG | 25 | | 0.391 | 38 | 0.521 |  |  |
|  | A allele | 37 | | 0.289 | 54 | 0.370 | 0.1605 |  |
|  | G allele | 91 | | 0.711 | 92 | 0.630 |  |  |
| rs9869263 | AA | 3 | | 0.048 | 4 | 0.056 | 0.6494 | synonymous codon |
|  | GG | 43 | | 0.683 | 43 | 0.606 |  |  |
|  | AG | 17 | | 0.270 | 24 | 0.338 |  |  |
|  | A allele | 23 | | 0.183 | 32 | 0.225 | 0.4494 |  |
|  | G allele | 103 | | 0.817 | 110 | 0.775 |  |  |
| rs10513846 | AA | 11 | | 0.172 | 13 | 0.181 | 0.8138 | intron variant |
|  | GG | 21 | | 0.328 | 20 | 0.278 |  |  |
|  | AG | 32 | | 0.500 | 39 | 0.542 |  |  |
|  | A allele | 54 | | 0.422 | 65 | 0.451 | 0.7135 |  |
|  | G allele | 74 | | 0.578 | 79 | 0.549 |  |  |
| rs10513847 | AA | 18 | | 0.281 | 21 | 0.288 | 0.9888 | intron variant |
|  | GG | 11 | | 0.172 | 13 | 0.178 |  |  |
|  | AG | 35 | | 0.547 | 39 | 0.534 |  |  |
|  | A allele | 71 | | 0.555 | 81 | 0.555 | 1 |  |
|  | G allele | 57 | | 0.445 | 65 | 0.445 |  |  |
| rs3774019 | AA | 10 | | 0.156 | 13 | 0.183 | 0.5216 | intron variant |
|  | GG | 18 | | 0.281 | 25 | 0.352 |  |  |
|  | AG | 36 | | 0.563 | 33 | 0.465 |  |  |
|  | A allele | 56 | | 0.438 | 59 | 0.415 | 0.8054 |  |
|  | G allele | 72 | | 0.563 | 83 | 0.585 |  |  |
| rs9835663 | CC | 10 | | 0.156 | 13 | 0.183 | 0.5216 | intron variant |
|  | TT | 18 | | 0.281 | 25 | 0.352 |  |  |
|  | CT | 36 | | 0.563 | 33 | 0.465 |  |  |
|  | C allele | 56 | | 0.438 | 59 | 0.415 | 0.8054 |  |
|  | T allele | 72 | | 0.563 | 83 | 0.585 |  |  |
| ***OCLN* (chromosome 5)** | | | | | | | | |
| rs28666036 | GG | 47 | | 0.734 | 58 | 0.806 | 0.1577 | upstream variant (2 kb) |
|  | TT | 3 | | 0.047 | 0 | 0.000 |  |  |
|  | GT | 14 | | 0.219 | 14 | 0.194 |  |  |
|  | G allele | 108 | | 0.844 | 130 | 0.903 | 0.1475 |  |
|  | T allele | 20 | | 0.156 | 14 | 0.097 |  |  |
| rs28407209 | CC | 1 | | 0.016 | 1 | 0.014 | 0.8754 | upstream variant (2 kb) |
|  | TT | 46 | | 0.719 | 49 | 0.681 |  |  |
|  | CT | 17 | | 0.266 | 22 | 0.306 |  |  |
|  | C allele | 19 | | 0.148 | 24 | 0.167 | 0.7406 |  |
|  | T allele | 109 | | 0.852 | 120 | 0.833 |  |  |
| ***NPC1L1* (chromosome 5)** | | | | | | | | |
| rs2072183 | CC | 11 | | 0.172 | 11 | 0.151 | 0.6908 | synonymous codon, upstream variant (2 kb) |
|  | GG | 27 | | 0.422 | 27 | 0.370 |  |  |
|  | CG | 26 | | 0.406 | 35 | 0.479 |  |  |
|  | C allele | 48 | | 0.375 | 57 | 0.390 | 0.8046 |  |
|  | G allele | 80 | | 0.625 | 89 | 0.610 |  |  |
| rs4720470 | CC | 26 | | 0.406 | 29 | 0.414 | 0.9909 | intron variant |
|  | TT | 8 | | 0.125 | 9 | 0.129 |  |  |
|  | CT | 30 | | 0.469 | 32 | 0.457 |  |  |
|  | C allele | 82 | | 0.641 | 90 | 0.643 | 1 |  |
|  | T allele | 46 | | 0.359 | 50 | 0.357 |  |  |
| rs1468384 | CC | 64 | |  | 73 |  |  | missense |
| rs202188870 | AG | 64 | |  | 73 |  |  | downstream variant (500 bp) |
| rs17655652 | TT | 63 | | 0.984 | 72 | 0.986 | 1 | upstream variant (2 kb) |
|  | CC | 0 | | 0.000 | 0 | 0.000 |  |  |
|  | CT | 1 | | 0.016 | 1 | 0.014 |  |  |
|  | T allele | 127 | | 0.992 | 145 | 0.993 | 1 |  |
|  | C allele | 1 | | 0.008 | 1 | 0.007 |  |  |
| ***APOE* (chromosome 19)** | | | | | | | | |
| rs769449 | AA | 0 | | 0.000 | 0 | 0.000 | 0.8325 | intron variant |
|  | GG | 50 | | 0.781 | 58 | 0.806 |  |  |
|  | AG | 14 | | 0.219 | 14 | 0.194 |  |  |
|  | A allele | 14 | | 0.109 | 14 | 0.097 | 0.8422 |  |
|  | G allele | 114 | | 0.891 | 130 | 0.903 |  |  |

*IRF3*, interferon regulatory factor 3; *IFNG*, interferon gamma; *IL4*, interleukin 4; *TBX21*, T-cell-specific T-box transcription factor; *IFNL3*, interferon lambda 3; *FOXP3*, forkhead box P3; *RORC*, RAR-related orphan receptor C; *TLR3*, toll-like receptor 3; *TLR7*, toll-like receptor 7; *TLR9*, toll-like receptor 9; *CXCR5*, C-X-C motif chemokine receptor 5; *ICOS*, inducible T cell co-stimulator; *IL21*, interleukin 21; *CD40LG*, CD40 ligand; *CXCL13*, C-X-C motif chemokine ligand 13; *BCL6*, B-cell CLL/lymphoma 6; *CD81*, CD81 molecule; *LDLR*, low density lipoprotein receptor; *SCARB1*, scavenger receptor class B type I; *CLDN1*, claudin 1; *OCLN*, occludin; *NPC1L1*, *NPC1*-like intracellular cholesterol transporter 1; *APOE*, apolipoprotein E.

SNP genotyping was conducted using the iPLEX MassARRAY system (Sequenom Inc., USA). Allele frequencies and genotype distributions for each SNP were calculated. Chi-square (χ^2^) and Fisher’s exact tests were used to evaluate the differences in SNP frequencies between HCV carriers and spontaneous resolvers. *P*-values, odds ratios (ORs), and 95% confidence intervals (95% CIs) were determined for association analysis. *P*-values (two-tailed) <0.05 were considered significant.

**Table S3.** *RORC* gene SNP allele frequencies in different populations

| Population | rs9826 | | | rs1521177 | | |
| --- | --- | --- | --- | --- | --- | --- |
|  | Allele frequencies | | Total count | Allele frequencies | | Total count |
|  | T | C |  | G | T |  |
| ACB | 0.8646 | 0.1354 | 192 | 0.2865 | 0.7135 | 192 |
| ASW | 0.7541 | 0.2459 | 122 | 0.4262 | 0.5738 | 122 |
| BEB | 0.8256 | 0.1744 | 172 | 0.1744 | 0.8256 | 172 |
| CEU | 0.6162 | 0.3838 | 198 | 0.5303 | 0.4697 | 198 |
| CLM | 0.5798 | 0.4202 | 188 | 0.6064 | 0.3936 | 188 |
| ESN | 0.9394 | 0.0606 | 198 | 0.2172 | 0.7828 | 198 |
| FIN | 0.6970 | 0.3030 | 198 | 0.3838 | 0.6162 | 198 |
| GBR | 0.7033 | 0.2967 | 182 | 0.4725 | 0.5275 | 182 |
| GIH | 0.7718 | 0.2282 | 206 | 0.2767 | 0.7233 | 206 |
| GWD | 0.8628 | 0.1372 | 226 | 0.3319 | 0.6681 | 226 |
| IBS | 0.6402 | 0.3598 | 214 | 0.4486 | 0.5514 | 214 |
| ITU | 0.8186 | 0.1814 | 204 | 0.1863 | 0.8137 | 204 |
| JPT | 0.5481 | 0.4519 | 208 | 0.4375 | 0.5625 | 208 |
| KHV | 0.7071 | 0.2929 | 198 | 0.2828 | 0.7172 | 198 |
| LWK | 0.8939 | 0.1061 | 198 | 0.3434 | 0.6566 | 198 |
| MSL | 0.8882 | 0.1118 | 170 | 0.2647 | 0.7353 | 170 |
| MXL | 0.6875 | 0.3125 | 128 | 0.4609 | 0.5391 | 128 |
| PEL | 0.6529 | 0.3471 | 170 | 0.4235 | 0.5765 | 170 |
| PJL | 0.6731 | 0.3269 | 208 | 0.4279 | 0.5721 | 208 |
| PUR | 0.7843 | 0.2157 | 204 | 0.2451 | 0.7549 | 204 |
| STU | 0.7843 | 0.2157 | 204 | 0.2451 | 0.7549 | 204 |
| TSI | 0.5888 | 0.4112 | 214 | 0.5093 | 0.4907 | 214 |
| YRI | 0.8843 | 0.1157 | 216 | 0.2731 | 0.7269 | 216 |
| CDX | 0.6075 | 0.3925 | 186 | 0.3495 | 0.6505 | 186 |
| CHB | 0.7184 | 0.2816 | 206 | 0.2379 | 0.7621 | 206 |
| CHS | 0.7238 | 0.2762 | 210 | 0.2333 | 0.7667 | 210 |
| **Primary cohort** | 0.7409 | 0.2591 | 274 | 0.2445 | 0.7555 | 274 |
| ***IFNL3* favorable sub-cohort** | 0.7521 | 0.2479 | 242 | 0.2438 | 0.7562 | 242 |

*P*-values (χ^2^ test) for comparisons between allele distributions in the primary cohort and *IFNL3* favorable sub-cohort with CDX, CHB, and CHS are provided below.

Primary cohort: rs9826, cohort vs. CHB (0.604), CHS (0.680), and CDX (0.003); rs1521177, cohort vs. CHB (0.914), CHS (0.830), and CDX (0.016).

*IFNL3* favorable sub-cohort: rs9826, sub-cohort vs. CHB (0.452), CHS (0.520), and CDX (0.002); rs1521177, sub-cohort vs. CHB (0.912), CHS (0.826), and CDX (0.017).

There were no significant differences in allele distributions between study cohorts and the CHB and CHS populations, while there were significant differences between study cohorts and CDX.

All the data are from <https://www.ncbi.nlm.nih.gov/variation/tools/1000genomes/> EXCEPT the study cohort. ACB, African Caribbeans in Barbados; ASW, Americans of African Ancestry in SW USA; BEB, Bengali from Bangladesh; CDX, Chinese Dai in Xishuangbanna, China; CEU, Utah Residents (CEPH) with Northern and Western Ancestry; CHB, Han Chinese in Beijing, China; CHS, Southern Han Chinese; CLM, Colombians from Medellin, Colombia; ESN, Esan in Nigeria; FIN, Finnish in Finland; GBR, British in England and Scotland; GIH, Gujarati Indian from Houston, Texas; GWD, Gambian in Western Divisions in the Gambia; IBS, Iberian Population in Spain; ITU, Indian Telugu from the UK; JPT, Japanese in Tokyo, Japan; KHV, Kinh in Ho Chi Minh City, Vietnam; LWK, Luhya in Webuye, Kenya; MSL, Mende in Sierra Leone; MXL, Mexican Ancestry from Los Angeles USA; PEL, Peruvians from Lima, Peru; PJL, Punjabi from Lahore, Pakistan; PUR, Puerto Ricans from Puerto Rico; STU, Sri Lankan Tamil from the UK; TSI, Toscani in Italia;

YRI , Yoruba in Ibadan, Nigeria.

**Table S4.** The Hardy–Weinberg Equilibrium (HWE) test of all SNPs in the whole study population.

| SNP | chi^2^ | Fisher's *P*-value |
| --- | --- | --- |
| *IFNL3* gene |  |  |
| rs12979860 | 1.709351 | 0.191130 |
| rs8099917 | 0.196568 | 0.657525 |
| rs12980275 | 1.281159 | 0.257738 |
| *RORC* gene |  |  |
| rs9826 | 0.284671 | 0.593680 |
| rs1521177 | 0.303708 | 0.581592 |

**Table S5.** Genotype and allele frequency distributions of *RORC* SNPs in HIV^pos^ group.

| SNP | Genotype | HIV^pos^ Chronic HCV^*^ (n=53) | HIV^pos^ Resolved HCV ^*^ (n=28) | P-value | OR (95% CI) |
| --- | --- | --- | --- | --- | --- |
| *RORC* gene |  |  |  |  |  |
| rs9826 | CC | 0 (0) | 0 (0) | 0.777 |  |
| (3′ UTR) | TT | 35 (0.66) | 18 (0.69) |  |  |
|  | CT | 18 (0.34) | 8 (0.31) |  |  |
|  | C allele | 18 (0.17) | 8 (0.15) | 0.799 | 1.04 (0.78–1.38) |
|  | T allele | 88 (0.83) | 44 (0.85) |  | 0.92 (0.49–1.73) |
|  |  |  |  |  |  |
| rs1521177 | GG | 0 (0) | 0 (0) | 0.980 |  |
| (intron) | TT | 38 (0.72) | 20 (0.73) |  |  |
|  | GT | 15 (0.28) | 8 (0.27) |  |  |
|  | G allele | 15 (0.14) | 8 (0.14) | 0.981 | 0.99 (0.72–1.38) |
|  | T allele | 91 (0.86) | 48 (0.86) |  | 1.01 (0.55–1.84) |

^*^Number of cases (frequency). SNP genotypes were determined using the iPLEX MassARRAY system (Sequenom Inc., USA), and allele frequencies and genotype distributions were calculated. Chi-square (χ^2^) and Fisher’s exact tests were used to evaluate differences in SNP frequencies between HCV carriers and spontaneous resolvers. *P*-values, odds ratios (ORs), and 95% confidence intervals (95% CIs) were determined for association analysis. *P*-values (two-tailed) <0.05 were considered significant.

**Table S6.** Genotype and allele frequency distributions of *RORC* SNPs in HIV^neg^ HCV carriers of the primary cohort.

| SNP | Genotype | HCV 2a^*^ (n=25) | HCV 1b^*^ (n=39) | P-value | OR (95% CI) |
| --- | --- | --- | --- | --- | --- |
| *RORC* gene |  |  |  |  |  |
| rs9826 | CC | 3 (0.36) | 4 (0.51) | 0.48 |  |
| (3′ UTR) | TT | 9 (0.52) | 20 (0.39) |  |  |
|  | CT | 13 (0.12) | 15 (0.10) |  |  |
|  | C allele | 21 (0.42) | 44 (0.56) | 0.115 | 0.70 (0.45–1.09) |
|  | T allele | 29 (0.58) | 34 (0.44) |  | 1.25 (0.95–1.67) |
|  |  |  |  |  |  |
| rs1521177 | GG | 3 (0.12) | 2 (0.05) | 0.238 |  |
| (intron) | TT | 9 (0.36) | 22 (0.46) |  |  |
|  | GT | 13 (0.52) | 15 (0.39) |  |  |
|  | G allele | 19 (0.38) | 19 (0.24) | 0.099 | 1.45 (0.95–2.23) |
|  | T allele | 31 (0.62) | 59 (0.76) |  | 0.76 (0.54–1.08) |

^*^Number of cases (frequency). SNP genotypes were determined using the iPLEX MassARRAY system (Sequenom Inc., USA), and allele frequencies and genotype distributions were calculated. Chi-square (χ^2^) and Fisher’s exact tests were used to evaluate differences in SNP frequencies between HCV carriers and spontaneous resolvers. *P*-values, odds ratios (ORs), and 95% confidence intervals (95% CIs) were determined for association analysis. *P*-values (two-tailed) <0.05 were considered significant.

**Table S7.** Linkage disequilibrium tests for *RORC* rs9826/rs1521177 in the primary cohort and the *IFNL3* favorable sub-cohort.

|  | Primary cohort | | | | *IFNL3* favorable sub-cohort | | | |
| --- | --- | --- | --- | --- | --- | --- | --- | --- |
|  | rs9826 | | rs1521177 | | rs9826 | | rs1521177 | |
|  | D′ | r^2^ | D′ | r^2^ | D′ | r^2^ | D′ | r^2^ |
| rs9826 | - | - | 0.831 | 0.639 | - | - | 0.812 | 0.645 |

D′ and r^2^ values were calculated using SHEsis software to evaluate the linkage disequilibrium between the *RORC* SNPs, rs9826 and rs1521177.
